# Supramolecular encapsulation of redox-active monomers to enable free-radical polymerisation†

**DOI:** 10.1039/d2sc02072f

**Published:** 2022-06-07

**Authors:** Stefan Mommer, Kamil Sokołowski, Magdalena Olesińska, Zehuan Huang, Oren A. Scherman

**Affiliations:** a Melville Laboratory for Polymer Synthesis, Yusuf Hamied Department of Chemistry, University of Cambridge Lensfield Road Cambridge CB2 1EW UK oas23@cam.ac.uk

## Abstract

Extended polymeric structures based on redox-active species are of great interest in emerging technologies related to energy conversion and storage. However, redox-active monomers tend to inhibit radical polymerisation processes and hence, increase polydispersity and reduce the average molecular weight of the resultant polymers. Here, we demonstrate that styrenic viologens, which do not undergo radical polymerisation effectively on their own, can be readily copolymerised in the presence of cucurbit[*n*]uril (CB[*n*]) macrocycles. The presented strategy relies on pre-encapsulation of the viologen monomers within the molecular cavities of the CB[*n*] macrocycle. Upon polymerisation, the molecular weight of the resultant polymer was found to be an order of magnitude higher and the polydispersity reduced 5-fold. The mechanism responsible for this enhancement was unveiled through comprehensive spectroscopic and electrochemical studies. A combination of solubilisation/stabilisation of reduced viologen species as well as protection of the parent viologens against reduction gives rise to the higher molar masses and reduced polydispersities. The presented study highlights the potential of CB[*n*]-based host–guest chemistry to control both the redox behavior of monomers as well as the kinetics of their radical polymerisation, which will open up new opportunities across myriad fields.

## Abstract 2

Extended polymeric structures based on redox-active species are of great interest in emerging technologies related to energy conversion and storage.

## Graphical abstract

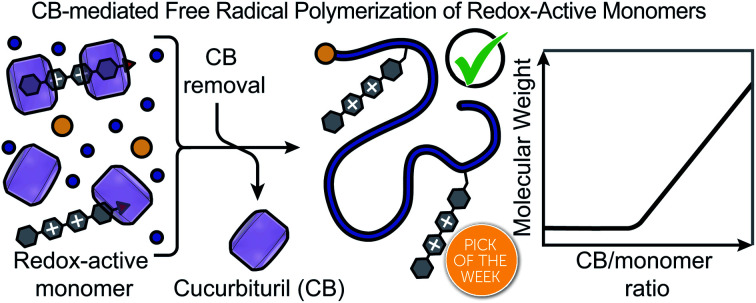

Polyviologens are redox-active polymers based on *N*-substituted bipyridinium derivatives which have emerged as promising materials for energy conversion and storage.^1–5^ Their physicochemical properties can be adjusted through copolymerisation of the redox-active viologen monomers.^6–8^ The resultant materials are stable, water soluble and exhibit fast electron transfer kinetics. Polyviologens have been commonly fabricated through step-growth polymerisation in linear and dendritic architectures,^9–13^ as supramolecular polymers,^14–16^ networks,^6,17,18^ and covalent organic frameworks.^19,20^ Alternatively, anionic/cationic or metathesis-based polymerisations are used to avoid interference of radical-stabilising monomers with the radical initiators, however, these techniques are highly water- and/or oxygen-sensitive.^21,22^ When free-radical polymerisation (FRP) is conducted in the presence of viologen species, its reduction can cause a depletion of active radicals and thus disruption of the polymerisation process. Despite varying solvents, comonomers and initiator loadings, the direct FRP of viologen-containing monomers remains therefore limited to molar masses of 30 kDa.^23–25^ Accessing higher molar masses has been possible *via* post-polymerisation modification,^26–28^ which has impacted the electrochemical properties of the resultant materials.^29,30^ Alternative strategies to access higher molar masses of redox-active polymers and control their polymerisation are highly desirable.

Incorporation of cucurbit[*n*]uril (CB[*n*]) macrocycles have lead to a variety of functional materials through host–guest chemistry.^31–34^ Moreover, the redox chemistry of viologens can be modulated through complexation with CB[*n*].^35–38^ Specifically, CB[*n*] (*n* = 7, 8) can tune the redox potential of pristine viologens and efficiently sequester monoreduced viologen radical cations, avoiding precipitation in aqueous environments. Further to this, we recently demonstrated that the viologen radical cation is stabilised by −20 kcal mol^−1^ when encapsulated in CB[7].^39^

Consequently, we envisioned that incorporating CB[*n*]s as additives prior to polymerisation could (i) overcome current limits in accessible molar masses, (ii) increase control over FRP of viologen-based monomers through encapsulation and (iii) enable separation of radical species avoiding aggregation.

Here, we demonstrate a new approach to control FRP of redox-active monomers leading to high molar masses and decreased dispersity of the resultant polymers. In absence of CB[*n*], co-polymerisation of the *N*-styryl-*N*′-phenyl viologen monomer 1^2+^ and *N*,*N*-dimethylacrylamide (DMAAm) only occurs at high initiator loadings (>0.5 mol%, Fig. 1a), leading to low molecular weights and high polydispersity. Using our synthetic approach, 1^2+^ is efficiently copolymerised with DMAAm in the presence of CB[*n*] (*n* = 7, 8) macrocycles resulting in control of the polymer molar mass across a broad range, 4–500 kDa (Fig. 1b). Finally, CB[*n*] are successfully removed from the polymer *via* competitive host–guest binding and dialysis. Spectroscopic and electrochemical studies revealed that solubilisation/stabilisation of the reduced species and/or shielding of the redox-active monomers from electron transfer processes was responsible for this enhancement.

**Fig. 1:**
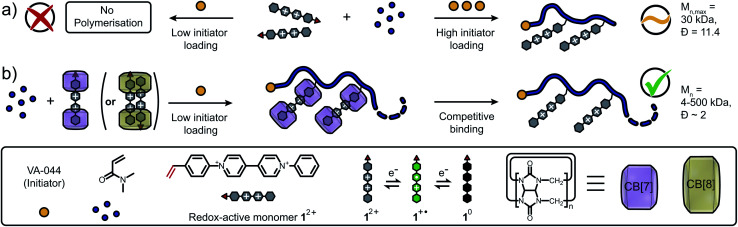
Schematic representation of the investigated polymerisation. (a) Conventional free radical polymerisation either completely fails to copolymerise redox-active monomers (low initiator loading) or delivers copolymers with limited molar masses and high dispersities (high initiator loading). (b) CB[*n*]-mediated protection suppresses interference of viologen monomers with radicals formed through the initiation process facilitating copolymerisation. The molar mass of the resulting copolymers is readily tunable *via* the amount of present CB[*n*] macrocycles and the CB[*n*] is post-synthetically removed *via* competitive binding to yield the final copolymer with desired molar mass. Cl^−^ counter-ions are omitted for clarity.

Recent studies on symmetric aryl viologens demonstrated 2 : 2 binding modes with CB[8] and high binding constants (up to *K*_a_ ∼ 10^11^ M^−2^).^40,41^ Incorporation of polymerisable vinyl moieties, in combination with the relatively static structure of their CB[*n*] host–guest complexes, was postulated to allow polymerisation without unfavorable side reactions. The asymmetric *N*-styryl-*N*′-phenyl viologen monomer 1^2+^ prepared for this study (Fig. S1a and S2–S13†) displays a linear geometry and was predicted to bind CB[*n*] (*n* = 7, 8) in a 2 : 1 and 2 : 2 binding fashion (Fig. S1b†).^40,42^ Binding modes between CB[*n*] (*n* = 7, 8) and 1^2+^ were investigated through titration experiments (^1^H NMR and ITC) which confirmed the formation of 1·(CB[7])_2_ and (1)_2_·(CB[8])_2_ (see Fig. S25 and S26†). ^1^H NMR titration of CB[7] with 1^2+^ demonstrates encapsulation of both aryl moieties (including the vinyl group) through upfield chemical shifts of the respective signals (Fig. 2a). Similar upfield shifts were observed for CB[8] (Fig. 2c). Different *para*-aryl substituents (vinyl *vs.* hydrogen) resulted in either head-to-tail or head-to-head (1)_2_·(CB[8])_2_ dimers (Fig. S1b and S26†), a previously reported phenomenon.^43^ Nonetheless, the reversible nature of the complex renders the vinyl group temporarily available for copolymerisation. In the presence of CB[8], 1^2+^ yields polymer molar masses of up to 500 kDa as its complexation is more robust. ITC data confirmed binding stoichiometry, with binding constants of *K*_a_ = 2.64 × 10^6^ M^−1^ for 1·(CB[7])_2_ and *K*_a_ = 9.02 × 10^10^ M^−2^ for (1)_2_·(CB[8])_2_ (Table S2, Fig. S29a and b†).

**Fig. 2:**
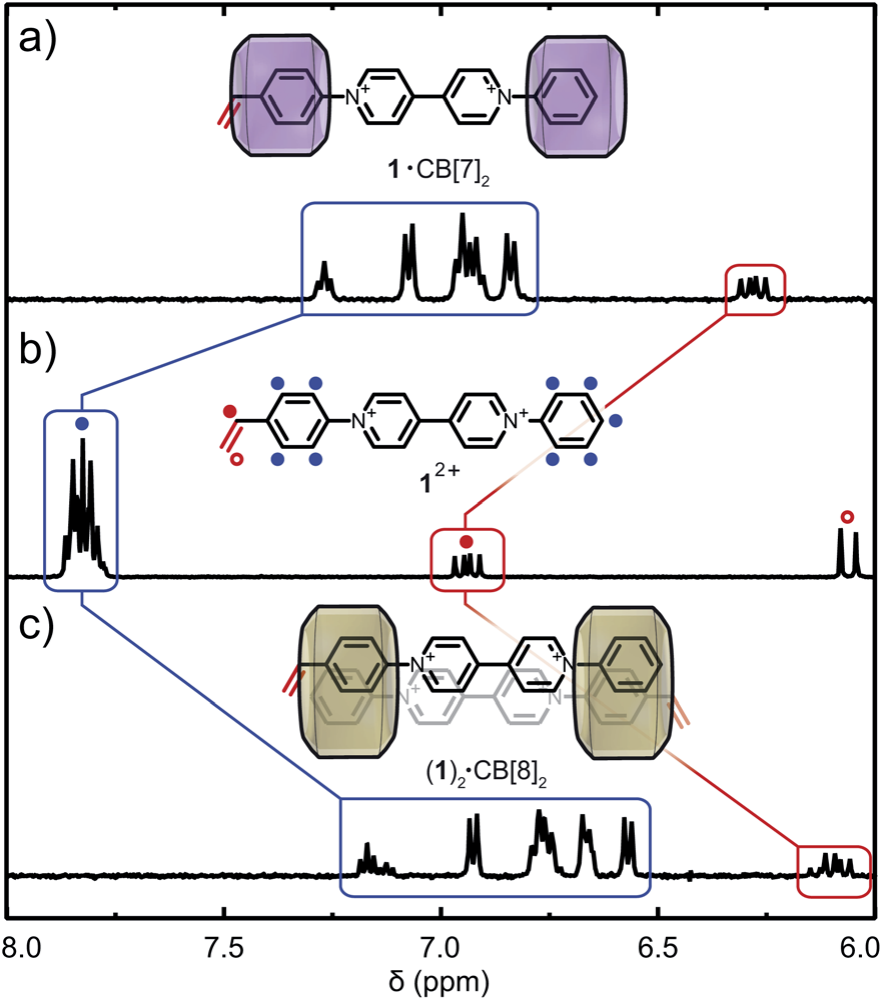
Supramolecular complexation of 1^2+^ and CB[*n*]. ^1^H NMR spectra of 1^2+^ at (a) *χ*_CB[7]_ = 2, (b) *χ*_CB[*n*]_ = 0 and (c) *χ*_CB[8]_ = 1 in D_2_O. Cl^−^ counter-ions are omitted for clarity.

The free radical copolymerisation of 1^2+^ and DMAAm ([M] = 2 M), in the absence of CB[*n*], was based on optimised DMAAm homopolymerisations (Fig. S14 and S15†) and full conversion was confirmed by ^1^H NMR spectroscopy (Table S1 and Fig. S16†). 1^2+^ was maintained at 1 mol% relative to DMAAm and by varying the radical initiator concentration molar masses of up to 30 kDa with broad dispersities (*Đ* = 11.4) were obtained (Fig. S17†). Lower initiator concentrations (<0.25 mol%) limited polymerisation (*M*_n_ = 3.7 kDa) and size exclusion chromatography elution peaks exhibited extensive tailing, suggesting that 1^2+^ engages in radical transfer processes.

To verify our hypothesis that CB[*n*] macrocycles can modulate the redox behavior of 1^2+^, FRP of 1^2+^ and DMAAm was conducted with varying amounts of CB[*n*] (*n* = 7, 8) (Fig. 3, S18 and S20†). Full conversion of all monomers including their successful incorporation into the polymer was verified *via*^1^H NMR spectroscopy and SEC (Fig. S18 and S21–S23†). Using CB[7], the molar mass of the copolymers was tunable between *M*_n_ = 3.7–160 kDa (Fig. 3b and S21a†). Importantly, in the presence of CB[8], a broad range of molar masses *M*_n_ = 3.7–500 kDa were accessible for 0 < *χ*_CB[8]_ < 1.2 (Fig. S20 and S21b†). Increasing the CB[*n*] (*n* = 7, 8) concentration caused dispersity values to converge to *Đ* = 2.2 (*χ*_CB[8]_ = 1.2, *χ* is the ratio of CB[*n*] to the redox-active monomer, Fig. S20†). The copolymers were purified by addition of adamantylamine (competitive binder) prior to dialysis to deliver CB[*n*]-free redox-active copolymers (Fig. S23†).

**Fig. 3:**
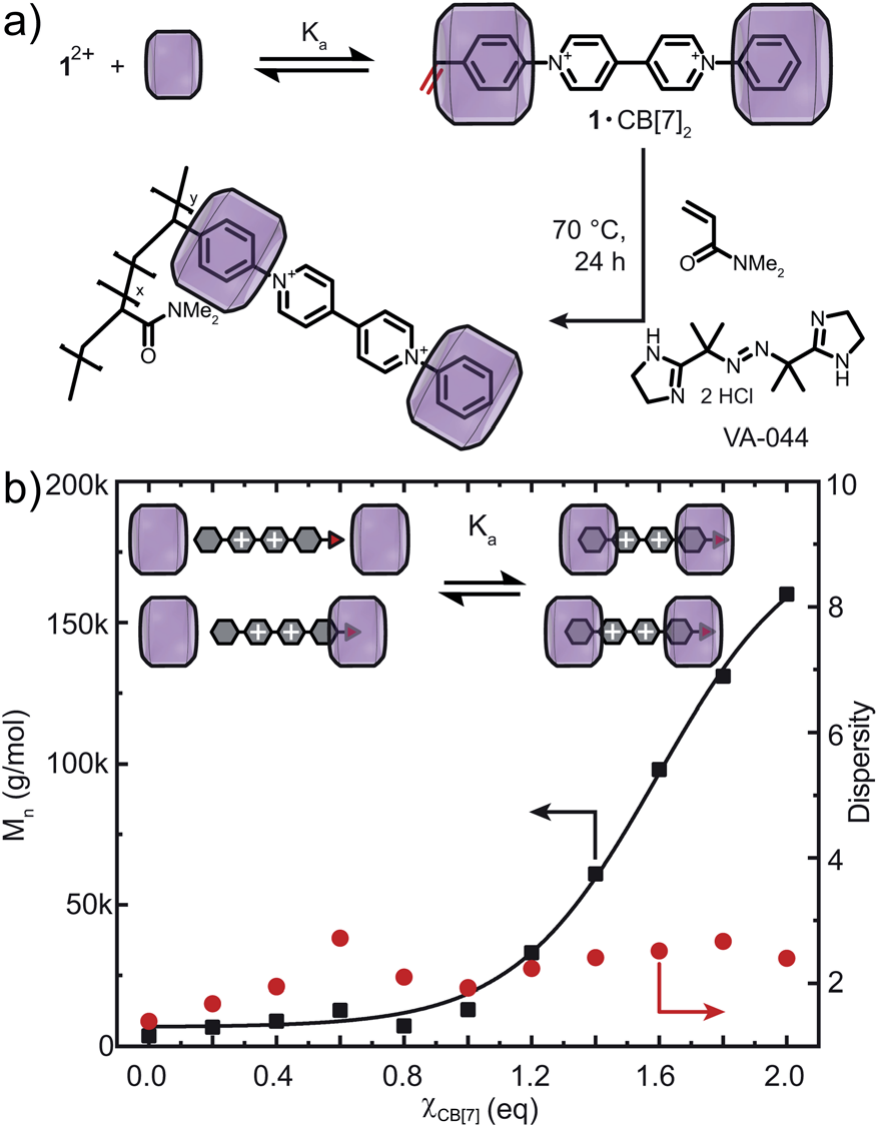
(a) *In situ* copolymerisation of DMAAm with 1^2+^ and CB[7]. (b) Molar mass and dispersity *vs.* amount of CB[7] in the system. Fitted curve is drawn to guide the eye. Cl^−^ counter-ions are omitted for clarity.

The range of molar masses obtainable through addition of CB[*n*] (*n* = 7, 8) correlated with the measured *K*_a_ (Fig. 3b and S20†). Binding of 1^2+^ to CB[8] was stronger and therefore lower concentrations of CB[8] were required to shift the binding equilibrium and mitigate disruption of the polymerisation. Dispersity values reached a maximum at *χ*_CB[7]_ = 0.6 or *χ*_CB[8]_ = 0.3, suggesting 1^+^˙ is only partially encapsulated. Consequently, higher CB[*n*] concentrations can enable FRP with lower initiator concentrations (0.10 mol%, Fig. S19†), which demonstrates the major role of complexation to modulate electron accepting properties of 1^2+^.

The redox-active monomer 1^2+^ can engage with propagating primary radicals (P_m˙_) to either be incorporated into the growing polymer chain (P_m_–1^2+^˙) or to abstract an electron deactivating it (P_m_). This deactivation likely occurs through oxidative termination producing 1^+^˙ (energetic sink), inactive oligo- and/or polymer chains (P_m_) and a proton H^+^, causing retardation of the overall polymerisation. Oxidative terminations have been previously observed in aqueous polymerisations of methyl methacrylate, styrenes and acrylonitriles that make use of redox initiator systems.^44–47^ Another example by Das *et al.* investigated the use of methylene blue as a retarder, with the primary radical being transferred to a methylene blue electron acceptor *via* oxidative termination, altogether supporting the outlined mechanism of our system (extended discussion see ESI, Section 1.4†).^48^

The process of retardation can, however, be successfully suppressed, when monomer 1^2+^ is encapsulated within CB[*n*] macrocycles. Herein the formation of 1·(CB[7])_2_ or (1)_2_·(CB[8])_2_ results in shielding of the redox-active component of 1^2+^ from other radicals within the system, hampering other electron transfer reactions. This inhibits termination and results in extended polymerisation processes leading to higher molar mass polymers through mitigation of radical transfer reactions. Moreover, suppressing the formation of 1^+^˙ through supramolecular encapsulation minimises both π and σ dimerisation of the emerging viologen radical species,^39^ preventing any further reactions that could impact the molar mass or polydispersity of the resulting polymers.

Cyclic voltammetry (CV) and UV-Vis titration experiments were conducted to provide insight into the impact of CB[*n*] on the redox behavior and control over FRP of 1^2+^. Excess of CB[*n*] (*n* = 7, 8) towards 1^2+^ resulted in a complete suppression of electron transfer processes (Fig. S31 and S32†). Initially, 1^2+^ shows a quasi-reversible reduction wave at −0.44 V forming 1^+^˙ (Fig. 4a). Increasing *χ*_CB[7]_, this reduction peak decreases and shifts towards more negative potentials (−0.51 V, *χ*_CB[7]_ = 1) accompanied by the formation of 1^2+^·(CB[7])_1_. A second cathodic peak emerges at −0.75 V due to the increased formation of 1^2+^·(CB[7])_2_. At *χ*_CB[7]_ = 2, this peak shifts to −0.80 V, where it reaches maximum intensity, once 1^2+^·(CB[7])_2_ is the dominating species in solution. When 2 < *χ*_CB[7]_ < 4, the intensity of the reduction peak decreases and the complexation equilibrium is shifted towards the bound state, complete suppression of the reduction peak occurs at *χ*_CB[7]_ = 4. Similarly, the oxidation wave intensity is reduced by 95% at *χ*_CB[7]_ = 4 causing suppression of potential oxidative radical transfer processes (Fig. 4c).

**Fig. 4:**
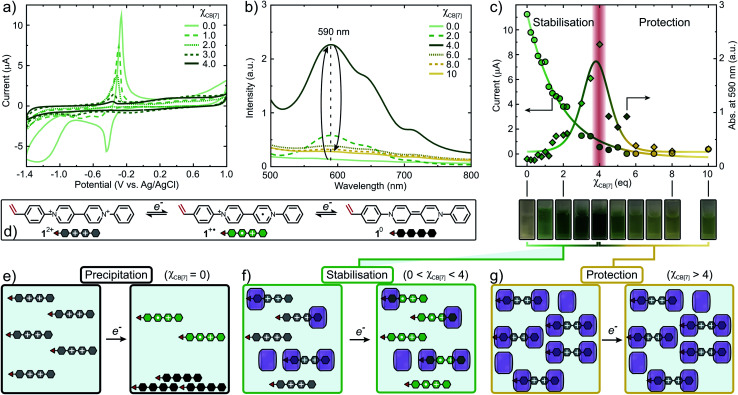
Mechanism of the CB[*n*]-mediated (*n* = 7, 8) strategy for the controlled copolymerisation of redox-active monomer 1^2+^. (a) Cyclic voltammogram with varying amounts of CB[7]. (b) UV-Vis titration of 1^2+^ with varying amounts of CB[7]. (c) Intensity decay of the oxidation peak at −0.27 V and change in absorption maximum of 1^+^˙ at 590 nm *vs. χ*_CB[7]_. (d) Electron transfer processes of 1^2+^ to generate 1^+^˙ and 1^0^. (e) Reduction of 1^2+^ resulting in precipitation of 1^0^. (f) Stabilisation of 1^+^˙ through encapsulation with CB[7]. (g) Protection of 1^2+^ from redox processes through CB[7]-mediated encapsulation.

The concentration of 1^+^˙ can be monitored using UV-Vis (Fig. 4b and S34†).^49^ Absorbance at 590 nm (*λ*_max_) *vs. χ*_CB[7]_ was plotted and the concentration of 1^+^˙ increases, reaching a maximum at *χ*_CB[7]_ = 4 (Fig. 4c). When *χ*_CB[7]_ > 4, a decrease in concentration of 1^+^˙ was observed. We postulate the following mechanism: at *χ*_CB[7]_ = 0, 1^2+^ is reduced to produce high concentrations of 1^+^˙ that partially disproportionates to form 1^0^, which precipitates (Fig. 4e and S34†). When 0 < *χ*_CB[7]_ < 4, increasing amounts of green 1^+^˙ are stabilised through encapsulation within CB[7] suppressing disproportionation (Fig. 4c (cuvette pictures), Fig. 4f). For *χ*_CB[7]_ > 4, 1^2+^ is protected from reduction through encapsulation (Fig. 4g).

To further demonstrate applicability of this strategy, we chose another viologen-based monomer 2^2+^ for copolymerisation (Fig. 5a). As opposed to 1^2+^, CB binds predominantly to the styryl moiety of 2^2+^ (Fig. S27 and S28†).^50^ ITC data showed that 2^2+^ binds CB[7] in a 1 : 1 fashion with a binding affinity of *K*_a_ = 2.32 × 10^6^ M^−1^ (Fig. S30 and Table S2†). Monomer 2^2+^ was also analysed *via* CV and showed three reversible reduction waves at −0.91 V, −0.61 V (viologen) and 0.40 V (styrene). Similar to 1^2+^, excess CB[7] selectively protects the molecule from redox processes, while the vinyl moiety remains accessible (Fig. 5a, S33c and d†). For CB[8], only partial suppression of electron transfer processes was observed (Fig. S33e and f†). We therefore chose CB[7] as an additive to increase control over FRP of 2^2+^ (Fig. 5b). Copolymerisation of 2^2+^ (1 mol%) and DMAAm ([M] = 2 M) at *χ*_CB[7]_ = 0 resulted in *M*_n_ = 28 kDa. When *χ*_CB[7]_ = 0.1, 0.2 or 0.3, *M*_n_ increased gradually from 124 to 230 and 313 kDa, respectively, demonstrating the potential of this strategy for FRP of redox-active monomers. Higher percentages of CB[7] led to copolymers with presumably higher molar masses causing a drastic decrease in solubility that prevented further analysis. Investigations on a broader spectrum of such copolymers, including those with higher contents of 2^2+^ are currently ongoing.

**Fig. 5:**
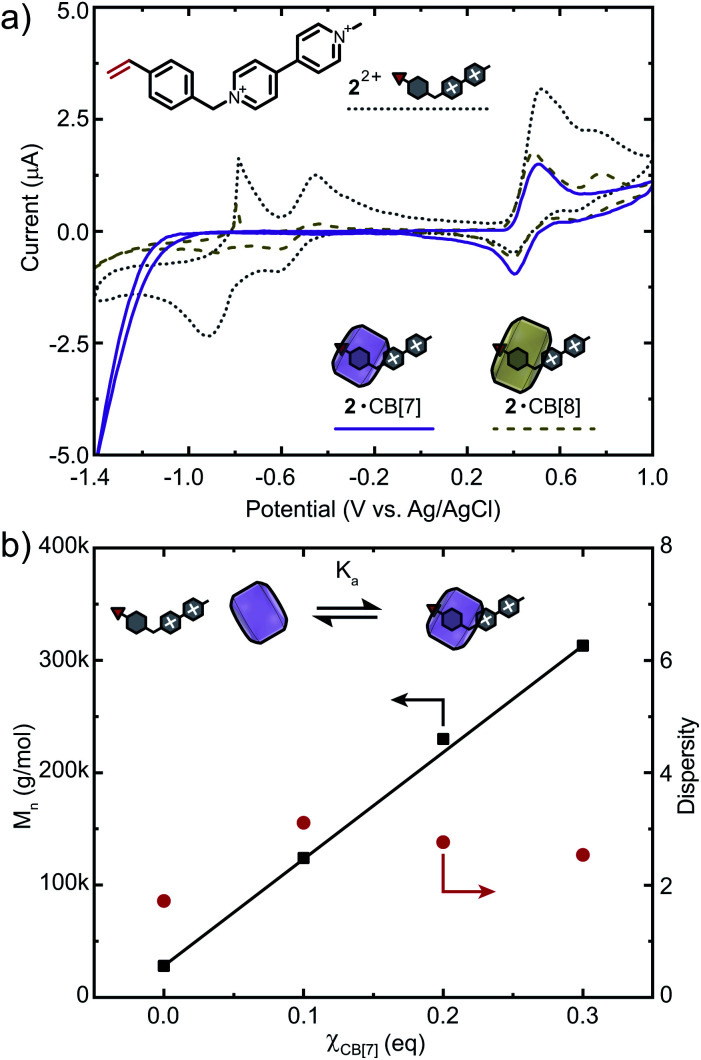
(a) Cyclic voltammogram of viologen-containing monomer 2^2+^ and its complexation with CB[*n*] (*n* = 7, 8) at a concentration of 1 mM using a scan rate of 10 mV s^−1^ in 0.1 mM NaCl solution. (b) Molar mass and dispersity of 2^2+^-containing copolymers *vs.* equivalents of CB[7]. Cl^−^ counter-ions are omitted for clarity.

In conclusion, we report a supramolecular strategy to induce control over the free radical polymerisation of redox-active building blocks, unlocking high molar masses and reducing polydispersity of the resulting polymers. Through the use of CB[*n*] macrocycles (*n* = 7, 8) for the copolymerisation of styrenic viologen 1^2+^, a broad range of molar masses between 3.7–500 kDa becomes accessible. Our mechanistic investigations elucidated that the redox behavior of monomer 1^2+^ is dominated by either CB[*n*]-mediated stabilisation of monoradical cationic species or protection of the encapsulated pyridinium species from reduction. In the stabilisation regime (*χ*_CB[7]_ < 4), 1^2+^ is reduced to form the radical cation 1^+^˙, which is subsequently stabilised through CB[7] encapsulation. Upon reaching a critical concentration of CB[7] (*χ*_CB[7]_ > 4), the system enters a protection-dominated regime, where reduction of 1^2+^ is suppressed and the concentration of 1^+^˙ diminishes. The resulting copolymers can be purified by use of a competitive binder to remove CB[*n*] macrocycles from the product. This strategy was successfully translated to a structurally different redox-active monomer that suffered similar limitations. We believe that the reported strategy of copolymerisation of redox-active monomers will open new avenues in the synthesis of functional materials for energy conversion and storage as well as for applications in electrochromic devices and (nano)electronics.

## Author contributions

Conceptualization: SM, KS, MO, OAS data curation: SM, KS, ZH, OAS formal analysis: SM, KS, ZH, OAS funding acquisition: OAS supervision: OAS writing – original draft: SM, KS, ZH writing – review & editing: SM, KS, OAS.

## Conflicts of interest

There are no conflicts to declare.

## Supplementary Material

SC-013-D2SC02072F-s001

## Data Availability

Data for this paper, including NMR, UV-Vis, CV and ITC are available at https://doi.org/10.17863/CAM.85780.
