# Supplementary material for: Supramolecular encapsulation of redox-active monomers to enable free-radical polymerisation
Source: Chem Sci. 2022 Jun 7;13(30):8791–6. doi: 10.1039/d2sc02072f (PMC9350630; doi:10.1039/d2sc02072f)
Supplement: SC-013-D2SC02072F-s001 [file SC-013-D2SC02072F-s001.pdf]

Electronic Supplementary Material (ESI) for Chemical Science.
This journal is © The Royal Society of Chemistry 2022

Supporting Information

Supramolecular Encapsulation of Redox-Active Monomers to Enable Free-Radical Polymerisation

Stefan Mommer, Kamil Sokołowski, Magdalena Olesińska, Zehuan Huang and Oren A. Scherman*

Melville Laboratory for Polymer Synthesis, Yusuf Hamied Department of Chemistry, University of Cambridge, Lensfield Road, CB12EW Cambridge, United Kingdom.

Table of contents

1 Experimental section 2
1.1 Materials 2
1.2 Methods 2
1.3 Synthesis 4
1.3.1 Synthetic Pathway towards Asymmetric Aryl,Aryl'-Functional Viologen **1**$^{2+}$ 4
1.3.2 1-(2,4-dinitrophenyl)-[4,4'-bipyridin]-1-ium chloride (**4**) 5
1.3.3 1-phenyl-[4,4'-bipyridin]-1-ium chloride (**5**) 6
1.3.4 1-(2,4-dinitrophenyl)-1'-phenyl-[4,4'-bipyridine]-1,1'-diium chloride (**6**) 7
1.3.5 1-phenyl-1'-(4-vinylphenyl)-[4,4'-bipyridine]-1,1'-diium chloride (**1**$^{2+}$) 11
1.3.6 General procedure for the free radical polymerisation of acrylamide monomers. 15
1.3.7 Synthesis of poly(DMAAm-*stat*-**1**) 16
1.3.8 General procedure for the CB[*n*]-mediated polymerisation of **1**$^{2+}$ and DMAAm 18
1.4 Extended discussion on CB[*n*] (*n*=7,8) assisted copolymerisation of DMAAm and **1**$^{2+}$ 22
1.5 NMR binding studies 23
1.6 ITC data 27
1.7 Cyclic Voltammetry 29
1.8 UV-Vis Experiments 32
2 References 33

1 Experimental section

1.1 Materials

1-Chloro-2,4-dinitrobenzene (ClDNB, 99%, Acros Organics), 4,4′-dipyridyl (98%, Sigma-Aldrich), aniline (>99%, Acros Organics), 4-vinylaniline (97%, Sigma-Aldrich), triethylamine (TEA, ≥99%, Sigma-Aldrich), dimethylacrylamide (DMAAm, 99%, Sigma-Aldrich), acrylamide (AAm, >98%, Sigma-Aldrich), 2,2'-azobis[2-(2-imidazolin-2-yl)propane]dihydrochloride (VA-044, 99%, TCI), 1-adamantylamine (ADA, >99%, Sigma-Aldrich) were used without further purification. Unless otherwise indicated, all solvents were purchased from commercial sources and were used without further purification. To remove residual inhibitor, DMAAm was treated with basic alumina prior use.

1.2 Methods

^{1}H and ^{13}C NMR spectra were recorded on a Bruker Avance III HD Spectrometer (400 MHz and 100 MHz, respectively) and are reported as follows: chemical shift δ (ppm) (multiplicity, coupling constant *J* (Hz), number of protons, assignment). Dimethylsulfoxide (DMSO, δ_H = 2.50 ppm, δ_C = 39.5 ppm) and D_2O (δ_H = 4.79 ppm) were used as an internal standard. Chemical shifts are reported in ppm to the nearest 0.01 ppm for ^{1}H and ^{13}C.

^{1}H NMR spectroscopic titrations were recorded on a Bruker AVANCE 500 with TCI Cryoprobe system (500 MHz) being controlled by TopSpin2.

Diffusion Ordered Spectroscopy (DOSY). The ^{1}H DOSY experiments were carried out using a modified version of the Bruker sequence ledbpgp2s involving, typically, 32 scans over 16 steps of gradient variation from 10% to 80% of the maximum gradient. Diffusion coefficients were evaluated in Dynamic Centre (a standard Bruker software) and determined by fitting the intensity decays according to the following equation:

$$I = I_0\, e^{\left[-D\gamma^2 g^2 \delta^2 \left(\Delta - \frac{\delta}{3}\right)\right]}$$

where I and I_0 represent the signal intensities in the presence and absence of gradient pulses respectively, D is the diffusion coefficient, $\gamma = 26753\, rad/s/Gauss$ is the ^{1}H gyromagnetic ratio, $\delta = 2.4\, ms$ is duration of the gradient pulse, $\Delta = 100\, ms$ is the total diffusion time and g is the applied gradient strength. The Monte Carlo simulation method was used for the error estimation of fitting parameters with a confidence level of 95%.

Molar masses (M_n and M_w) and dispersity values ($Đ = M_w/M_n$) were determined by size exclusion chromatography (SEC). SEC analyses were carried out with DMF as eluent. SEC with DMF (HPLC grade, VWR) as eluent was performed using a Shimadzu LC system equipped with a diode-array detector (SPD-M20A DAD) and a refractive index detector (RI, Optilab rEX, Wyatt). The eluent

contained 1 g/L LiBr (98%, Sigma-Aldrich). One precolumn (50x8 mm) and three PSS GRAM gel columns (300x8 mm) were applied at a flow rate of 1.0 $mL \cdot min^{-1}$ at 40 °C. The diameter of the gel particles measured 10 μm, the nominal pore widths were 100, 3000, and 3000 Å. Calibration was achieved using narrowly distributed poly(methyl methacrylate) standards.

Infrared spectra were measured on a Spectrum 100 FT-IR spectrometer (PerkinElmer) using an ATR Sampling Accessory unit (Perkin Elmer). Absorbance maxima are reported in wavenumbers (cm^{-1}) and only selected intensities are reported.

HRMS/ESI mass spectra were recorded on a Xevo G2-S ASAP spectrometer (Waters) and/or on a Thermo Scientific LTQ Orbitrap XL spectrometer.

Melting points were determined using an Electrothermal™ IA9000 Series Melting Point Apparatus (FisherScientific).

Semi-preparative HPLC was used for product purification using a Varian-940-LC and a KINETEX® EVO C18 column with a 5 μm pore size, a 100 Å particle size and dimensions of 150 x 21.2 mm. A mixture of water containing 0.1% TFA and acetonitrile or methanol was used as eluent; different gradients with a flow of 10 mL/min were applied.

Cyclic voltammetry (CV) experiments were carried out at 298 K in nitrogen purged 0.1 M $NaCl_{(aq)}$ solutions using a Metrohm Eco Chemie Autolab PGSTAT12 potentiostat, working on GPES 4.9 software. The following three-electrode configuration was used: a 3 mm glassy carbon as a working electrode, a platinum wire as the counter electrode and RE-5B Ag/AgCl BASI as a reference electrode. The glassy carbon electrode was polished before each measurement with 1 μm, 0.3 μm, and 0.05 μm alumina-H_2O slurry, respectively. CV was performed at variable scan rates ranging from 200 mV/s to 10 mV/s. The analyte concentration was set to 1 mM and up to 15 scans with an electric current range of 1 V to -1.4 V were run per measurement.

ITC measurements were performed on a Microcal AutoITC$_{200}$ in Milli-Q water or sodium acetate buffer (pH = 4.7) at 298 K. The guest molecules (monomers) with a twenty times higher concentration were titrated into a calibrated solution of host molecules (CB[8]). The calibration of the CB[8]-solution was carried out by a titration with 1-adamantylamine as a standard. For all experiments, a control titration in which the guest was titrated into the solvent without CB[8] was performed to check the heat of dilution, which emerged as minor in all cases. An experiment consisted of 20 to 40 consecutive injections of 1-2 μL with 60 s gap time between each injection. The first data point was removed before curve fitting. The experiments were carried out using a reference power of 6 μcal/s or 10 μcal/s. In

order to avoid bias or arbitrary offsets due to manual baseline adjustment, raw data were integrated by NITPIC, fitted in SEDPHAT or Origin 7.0 and visualised with GUSSI.[1]

UV/Vis Spectroscopy was carried out on a Varian Cary 400 UV/Vis spectrophotometer using a cuvette with 1 cm path length at room temperature (~20-25 °C). Analyte solutions were tested in the concentration of 1 mM or 0.1 mM.

1.3 Synthesis

1.3.1 Synthetic Pathway towards Asymmetric Aryl,Aryl'-Functional Viologen $\mathbf{1}^{2+}$

Reaction of viologen **3** and 1-chloro-2,4-dinitrobenzene (ClDNB) leads to asymmetric Zincke salt **4** in 90% yield.[2] Aromatic substitution of the activated Zincke salt **4** using aniline subsequently delivers phenyl-functional viologen **5** in quantitative yield. The remaining pyridyl nitrogen exhibits strongly reduced nucleophilicity and thus, a large excess of ClDNB is needed to give Zincke salt **6**. Finally, aromatic displacement of activated Zincke salt **6** using vinyl aniline yields styrene-functional monomer $\mathbf{1}^{2+}$ (Figure S2-13).

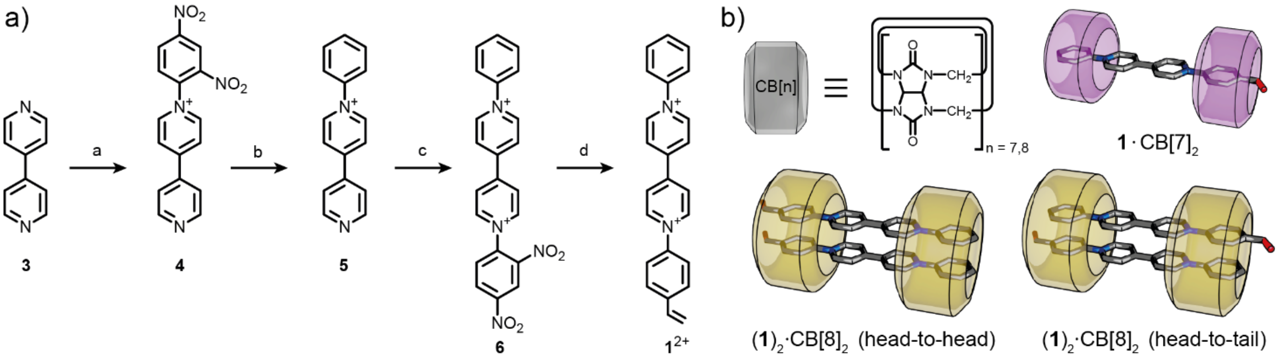

Figure S1. a) Synthetic pathway towards guest monomer $\mathbf{1}^{2+}$ carrying one vinyl group for subjection to polymerisation: (a) ClDNB (1.0 equiv.), 90 °C, 24 h, acetone; (b) aniline (3.0 equiv.), 90 °C, 2 h, EtOH; (d) ClDNB (20 equiv.), 90 °C, 48 h, NMP; (d) functional aniline (3.0 equiv.), TEA (1 mol%), 25 °C, 1 h, NMP. b) Schematic illustration of CB[*n*]-mediated complexes using guest monomer $\mathbf{1}^{2+}$, colour code: vinyl bond (red), pyridyl nitrogen (blue), CB[7] macrocycle (purple), CB[8] macrocycle (yellow). Chloride counter-ions are omitted for clarity.

1.3.2 1-(2,4-dinitrophenyl)-[4,4'-bipyridin]-1-ium chloride (**4**)

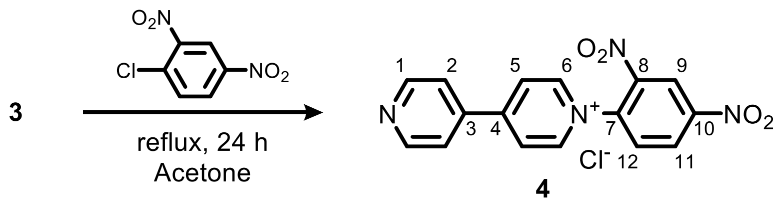

ClDNB (2.11 g, 10.4 mmol) was added to a stirred solution of 4,4'-dipyridyl **3** (1.62 g, 10.4 mmol) in acetone (10.4 mL, c = 1 M) at 25 °C. The reaction mixture was stirred and heated at reflux for 24 h. Next, the resulting precipitate was filtered off and washed with copious amounts of Et_2O to give **4** (3.46 g, 93%) as a light brown solid.

^{1}H NMR (400 MHz, DMSO-d_6) δ 9.54 (d, J = 7.0 Hz, 2H, H6), 9.16 (d, J = 2.5 Hz, 1H, H9), 9.00 (dd, J = 8.7, 2.5 Hz, 1H, H11), 8.97 – 8.92 (m, 4H, H2/H5), 8.44 (d, J = 8.7 Hz, 1H, H12), 8.18 (d, J = 6.2 Hz, 2H, H1). Spectroscopic data consistent with those reported in the literature.[3]

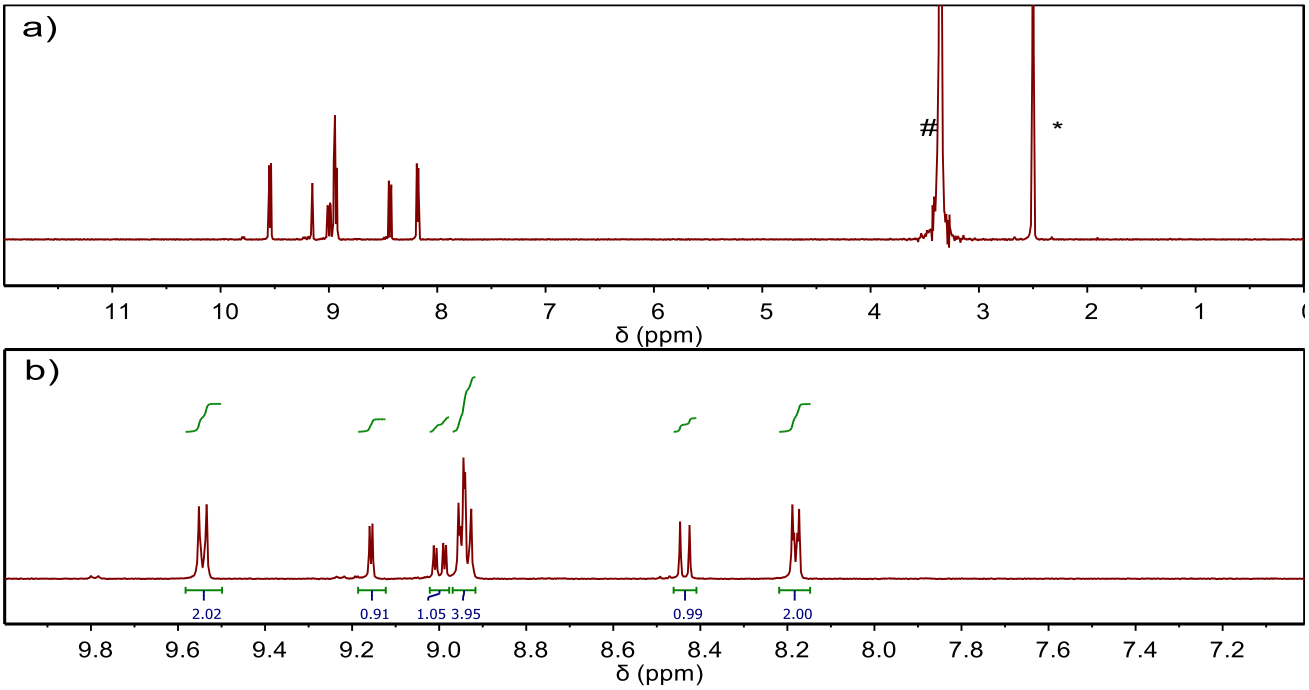

Figure S2. Full (a) and expanded (b) ^{1}H NMR spectrum of compound **4**. Residual solvent peaks: DMSO-d_6 (*), H_2O (#).

1.3.3 1-phenyl-[4,4'-bipyridin]-1-ium chloride (**5**)

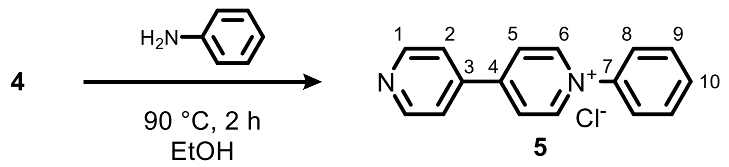

Aniline (763 *µ*L, 8.36 mmol) was added to a stirred solution of **4** (1.00 g, 2.79 mmol) in ethanol (28 mL, c = 0.1 M) at 90 °C. The reaction mixture was stirred and heated at reflux for 2 h. Next, the solvent was evaporated under reduced pressure to give the crude product. Purification by precipitation from methanol into Et_2O (1 L) gave product **5** (quant.) as a light brown solid. ^{1}H NMR (400 MHz, DMSO-*d*$_6$) δ 9.51 (d, *J* = 6.9 Hz, 2H, H6), 8.91 (d, *J* = 6.2 Hz, 2H, H1), 8.79 (d, *J* = 7.0 Hz, 2H, H5), 8.15 (d, *J* = 6.3 Hz, 2H, H2), 7.96 – 7.91 (m, 2H, H8), 7.81 – 7.75 (m, 3H, H9/H10). Spectroscopic data consistent with those reported in the literature.[4]

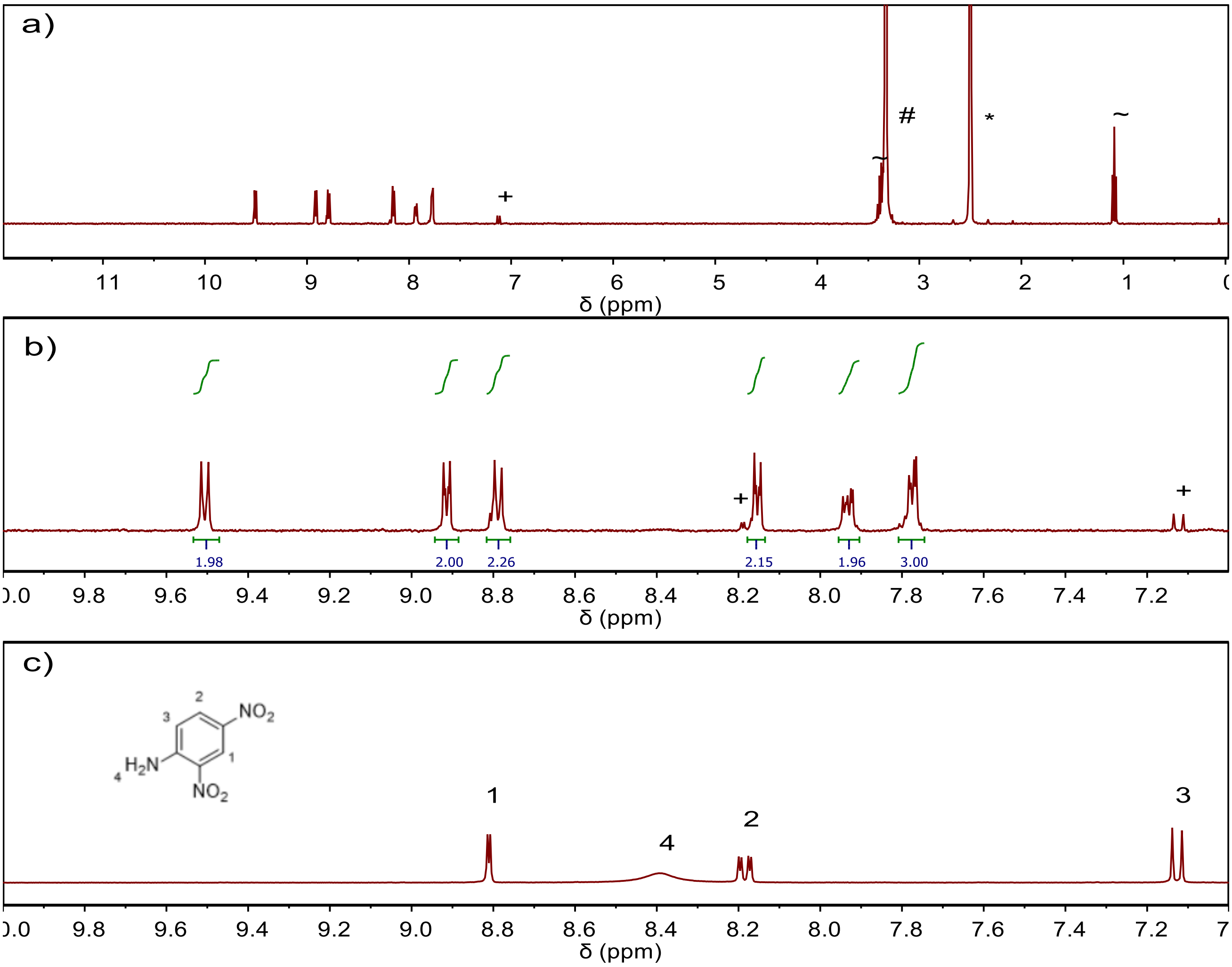

Figure S3. Full (a) and expanded (b) ^{1}H NMR spectrum of compound **5**. Residual solvent peaks: DMSO-*d*$_6$ (*), H_2O (#), Et_2O (~), trace impurity (+). c) ^{1}H NMR spectrum of the isolated trace impurity 2,4-dinitroaniline, a by-product of the aromatic substitution on the activated Zincke salt **4**.

1.3.4 1-(2,4-dinitrophenyl)-1'-phenyl-[4,4'-bipyridine]-1,1'-diium chloride (**6**)

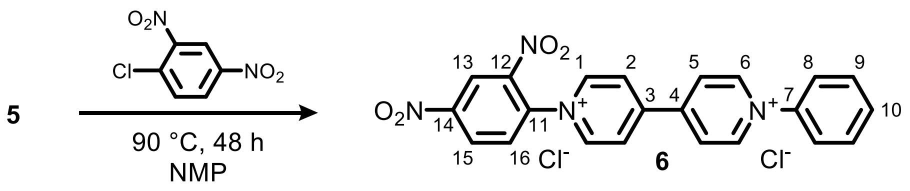

ClDNB (10.8 g, 53.4 mmol) was added to a stirred suspension of **5** (957 mg, 2.67 mmol) in NMP (5.34 mL, c = 0.5 M) at 90 °C. The reaction mixture was stirred and heated at 90 °C for 48 h. Purification by precipitation from the reaction mixture into MeCN (480 mL) gave product **6** (927 mg, 74%) as a light brown solid. mp. 220 °C. ^{1}H NMR (500 MHz, DMSO-d_6) δ 9.58 (d, J = 7.1 Hz, 2H, H1), 9.52 (d, J = 7.0 Hz, 2H, H6), 9.13 (d, J = 2.5 Hz, 1H, H13), 8.97 (d, J = 7.1 Hz, 2H, H2), 8.90 (dd, J = 8.7, 2.5 Hz, 1H, H15), 8.87 (d, J = 7.1 Hz, 2H, H5), 8.34 (d, J = 8.6 Hz, 1H, H16), 7.93 – 7.88 (m, 2H, H8), 7.79 – 7.75 (m, 3H, H9/10). ^{13}C NMR (126 MHz, DMSO-d_6) δ 152.41 (C3), 150.05 (C14), 149.85 (C4), 147.56 (C1), 146.29 (C6), 143.37 (C12), 142.89 (C7), 138.81 (C11), 132.60 (C10), 132.21 (C16), 131.25 (C9), 131.03 (C15), 127.79 (C5), 127.44 (C2), 125.16 (C8), 122.58 (C13). FT-IR (ATR) 3021, 2973, 1639, 1613, 1548, 1490, 1440, 1286, 1259, 1250, 1230, 1164, 1080, 1061, 1038, 915, 892, 838, 827, 771, 751, 719, 702, 692 cm^{-1}. ESI-HRMS m/z (%) 200.05851 (100) [$C_{22}H_{16}N_4O_4^{2+}$], 401.1248 (25) [$C_{22}H_{17}N_4O_4^{+}$].

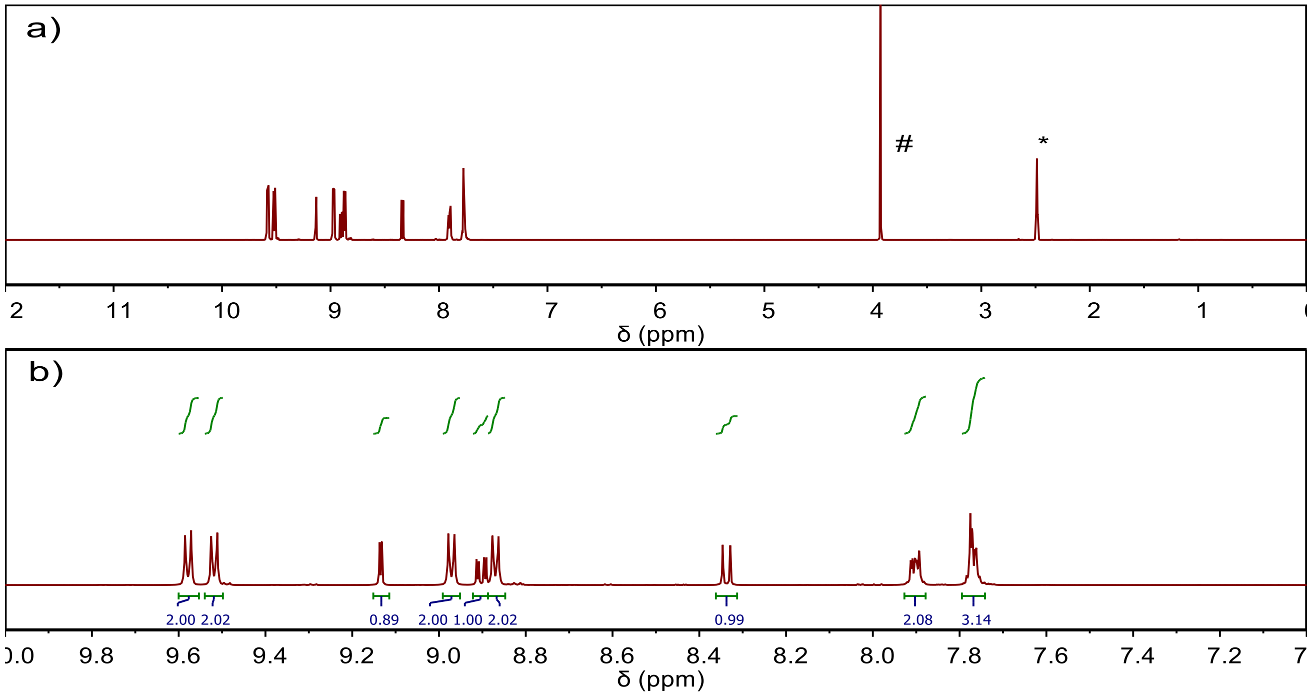

Figure S4. Full (a) and expanded (b) ^{1}H NMR spectrum of compound **6**. Residual solvent peaks: DMSO-d_6 (*), H_2O (#).

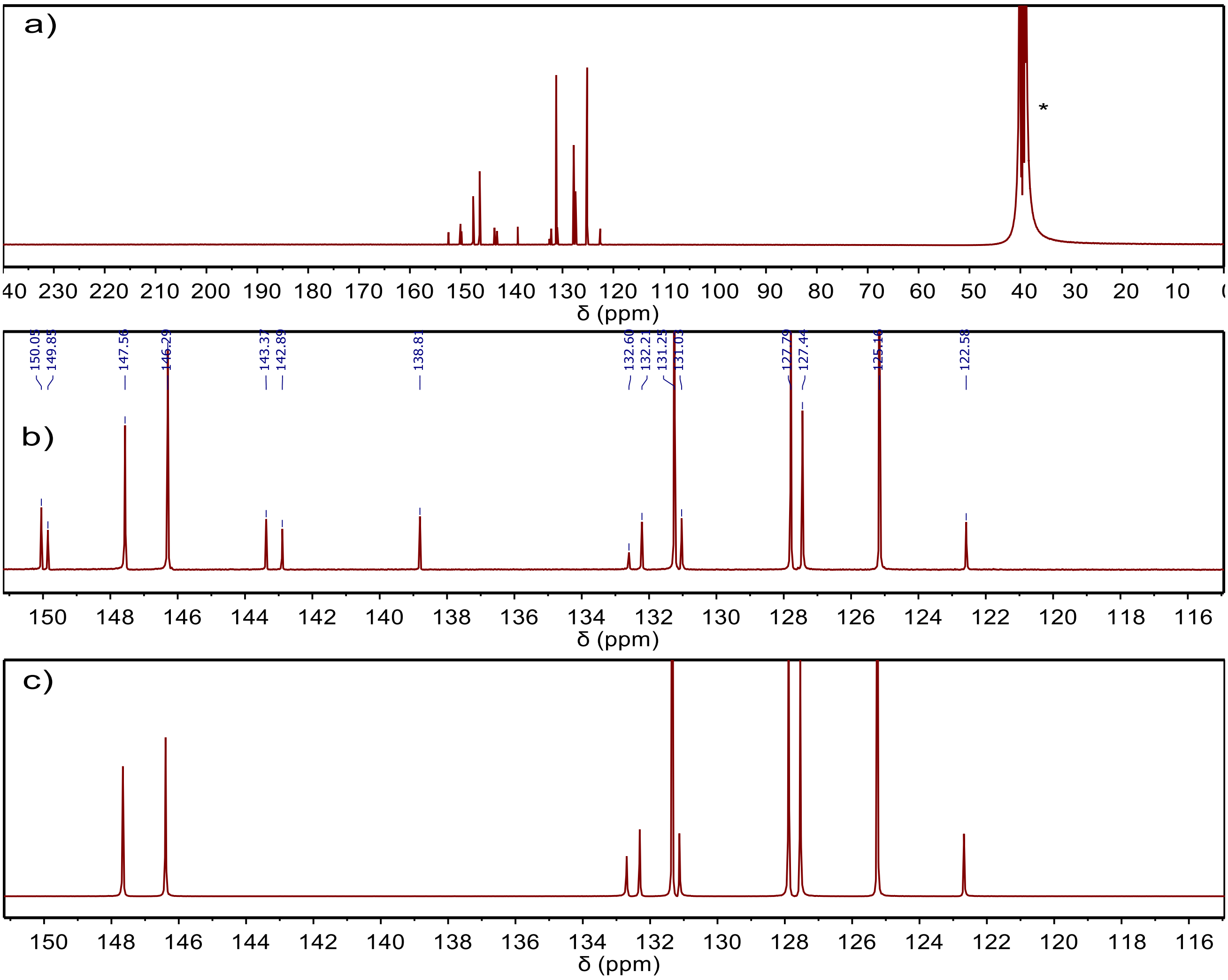

Figure S5. Full (a), expanded (b) and DEPT135 (c) ^{13}C NMR spectrum of compound **6** in DMSO-d_6 (*).

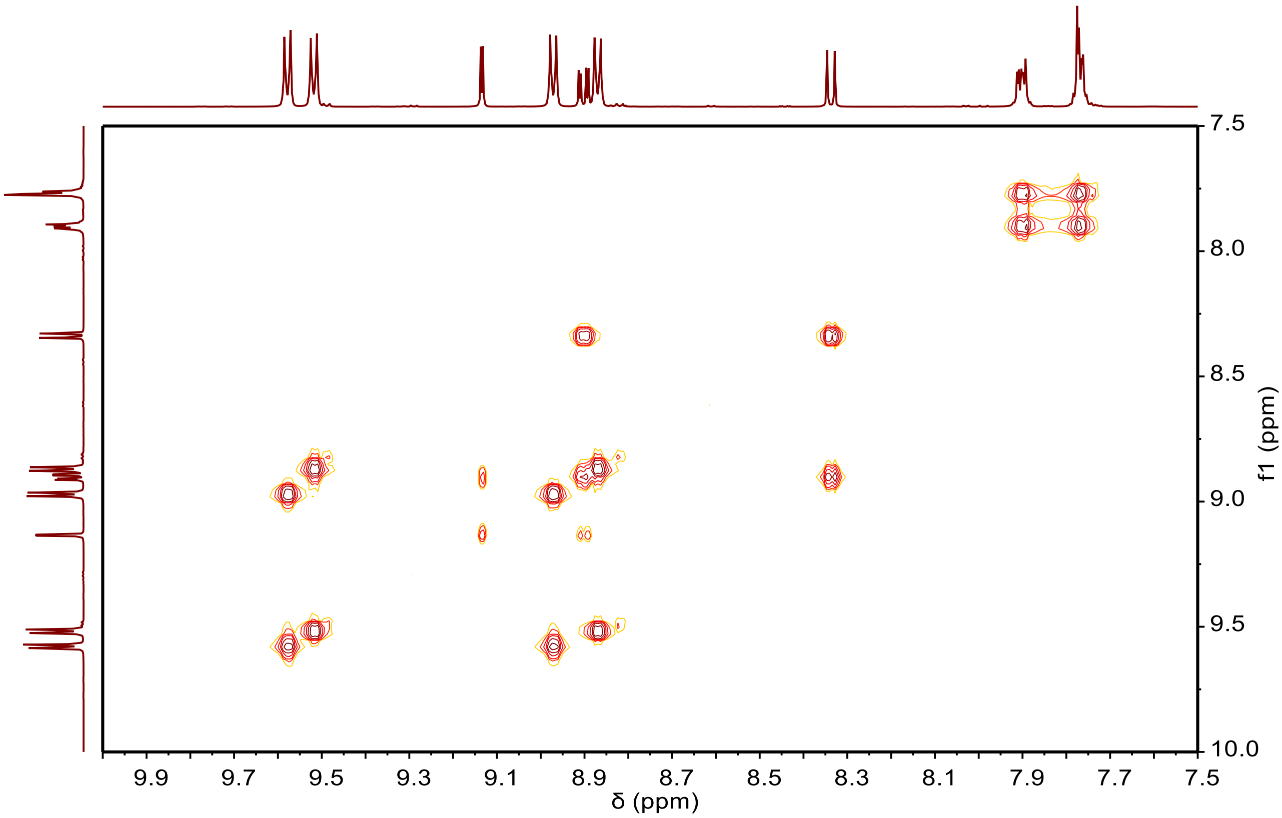

Figure S6. $^{1}H,^{1}H$-COSY NMR spectrum of **6**.

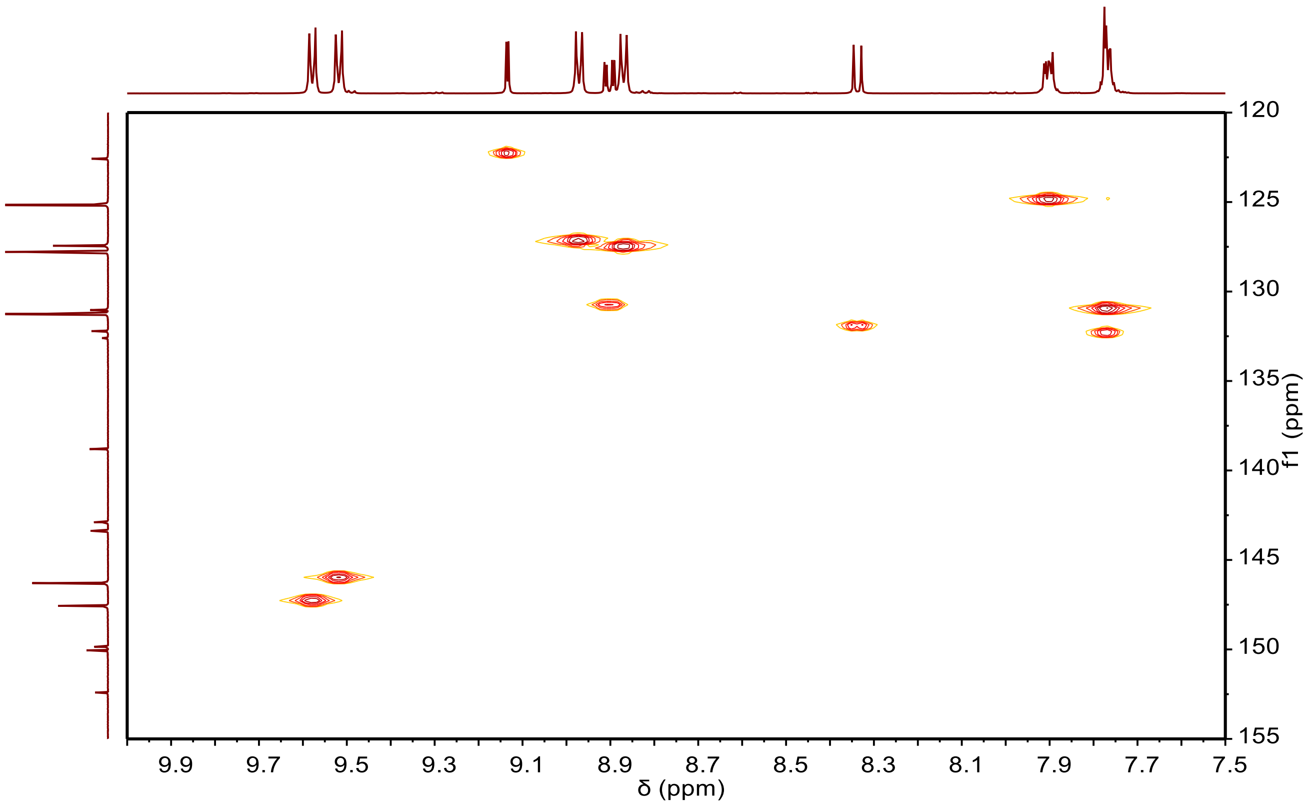

Figure S7. $^{1}H,^{13}C$-HSQC NMR spectrum of **6**.

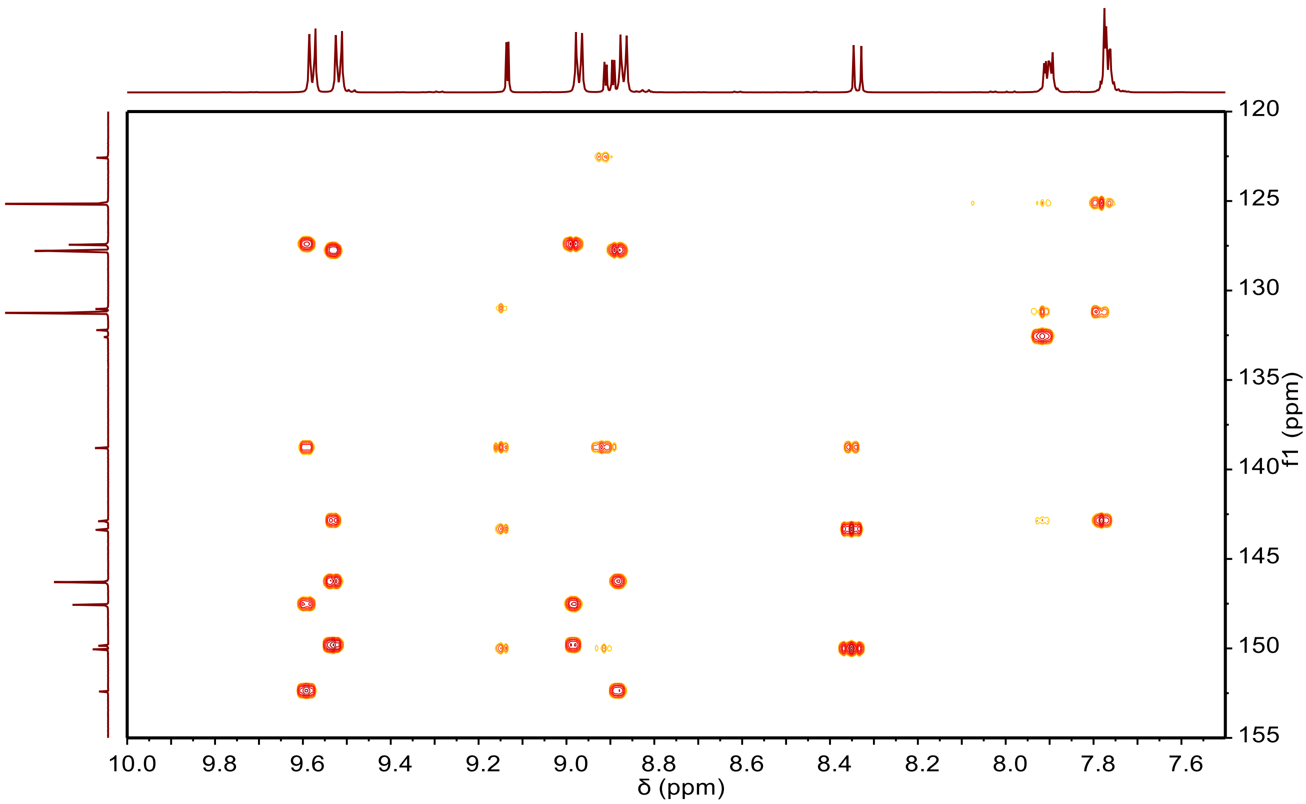

Figure S8. $^{1}H,^{13}C$-HSQC NMR spectrum of **6**.

1.3.5 1-phenyl-1'-(4-vinylphenyl)-[4,4'-bipyridine]-1,1'-diium chloride ($\mathbf{1}^{2+}$)

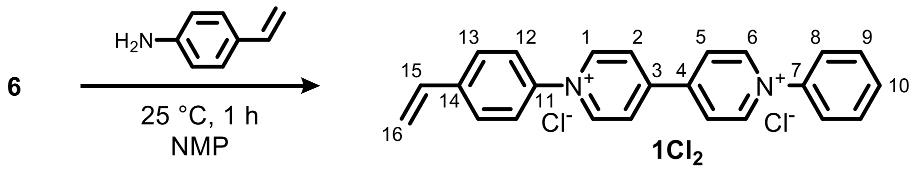

Vinylaniline (215 μL, 1.84 mmol) and triethylamine (0.85 μL, 6.12E-3 mmol) was added to a stirred suspension of **6** (288 mg, 0.612 mmol) in NMP (6.1 mL, c = 0.1 M) at rt. The reaction mixture was stirred at room temperature for 1 h. Purification by precipitation from the reaction mixture into MeCN (290 mL) gave product **1Cl₂** (236 mg, 95%) as a light brown solid. Further purification was achieved *via* semi-preparative HPLC using a gradient of 2% to 50% MeOH in H_2O (+ 0.1% TFA) over 20 min. mp. 118 °C. ^{1}H NMR (400 MHz, DMSO-d_6) δ 9.80 – 9.61 (m, 4H, H1/H6), 9.19 – 9.00 (m, 4H, H2/H5), 8.06 – 7.95 (m, 4H, H8/H12), 7.91 (d, J = 8.4 Hz, 2H, H13), 7.87 – 7.76 (m, 3H, H9/H10), 6.93 (dd, J = 17.6, 11.0 Hz, 1H, H15), 6.12 (d, J = 17.6 Hz, 1H, H16), 5.52 (d, J = 11.0 Hz, 1H, H16). ^{13}C NMR (101 MHz, DMSO-d_6) δ 148.98 (C4), 148.86 (C3), 146.03 (C1), 145.76 (C6), 142.30 (C7), 141.34 (C11), 140.34 (C14), 134.97 (C15), 131.66 (C10), 130.30 (C9), 127.69 (C13), 126.65 (C2/C5), 125.16 (C12), 124.90 (C8), 118.01 (C16). FT-IR (ATR) 3134, 3108, 3073, 3051, 1783, 1724, 1638, 1594, 1546, 1493, 1442, 1395, 1189, 1135, 930, 840, 798, 788, 769, 706 cm^{-1}. ESI-HRMS *m/z* (%) 168.08100 (100) [$C_{24}H_{20}N_2^{2+}$].

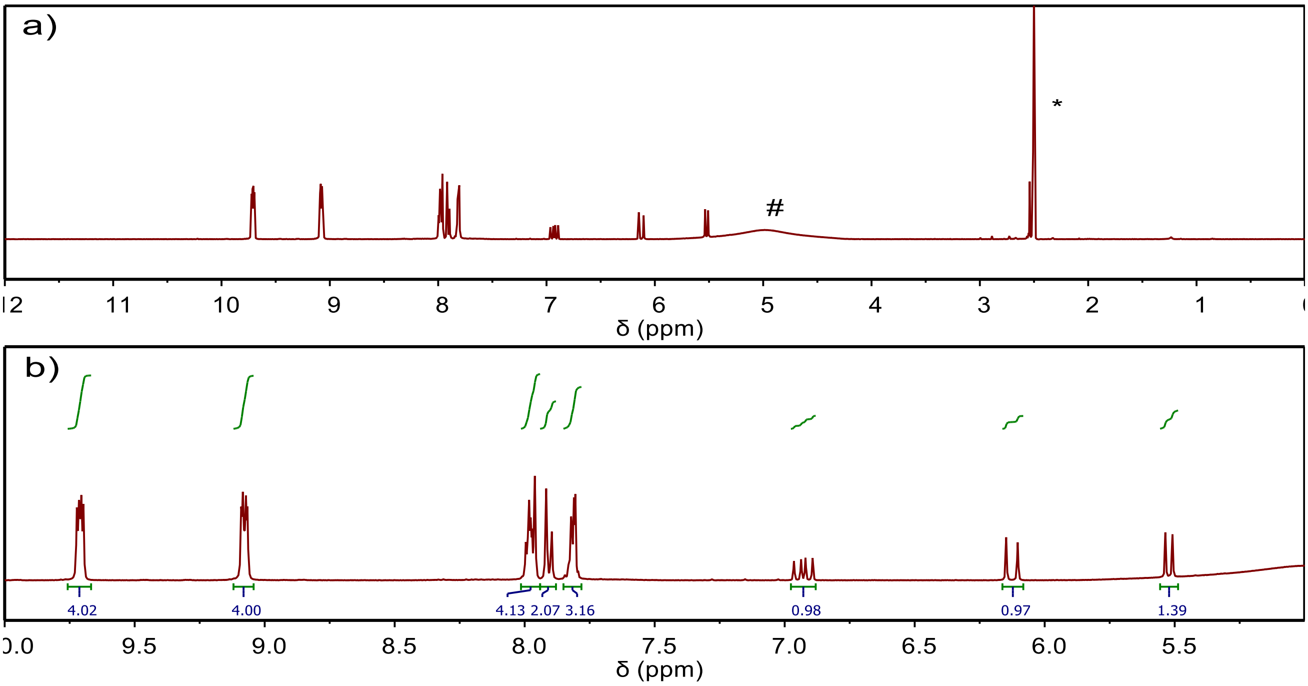

Figure S9. Full (a) and expanded (b) ^{1}H NMR spectrum of compound **1Cl₂**. Residual solvent peaks: DMSO-d_6 (*), H_2O (#).

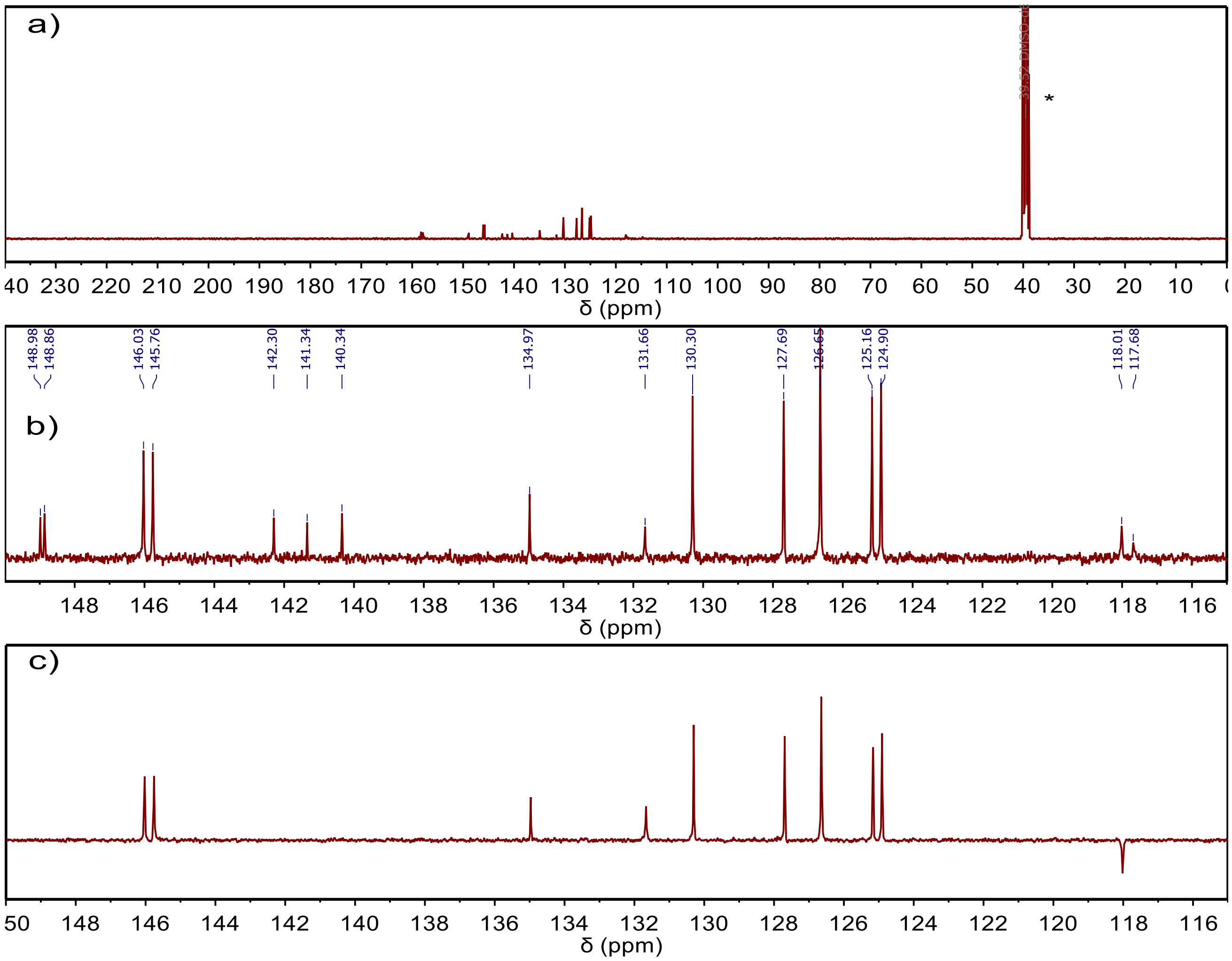

Figure S10. Full (a), expanded (b) and DEPT135 (c) ^{13}C NMR spectrum of compound **1Cl₂** in DMSO-*d*$_6$ (*).

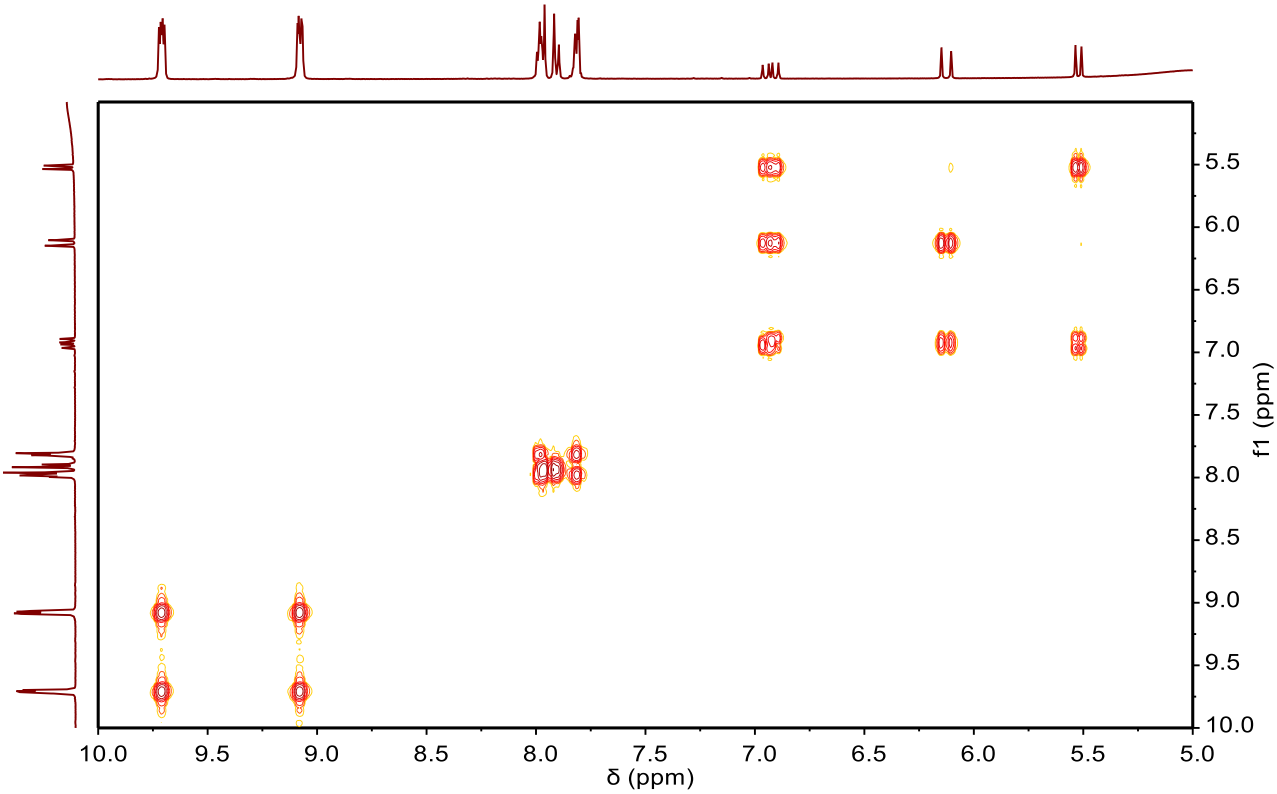

Figure S11. ^{1}H,^{1}H-COSY NMR spectrum of **$1Cl_2$**.

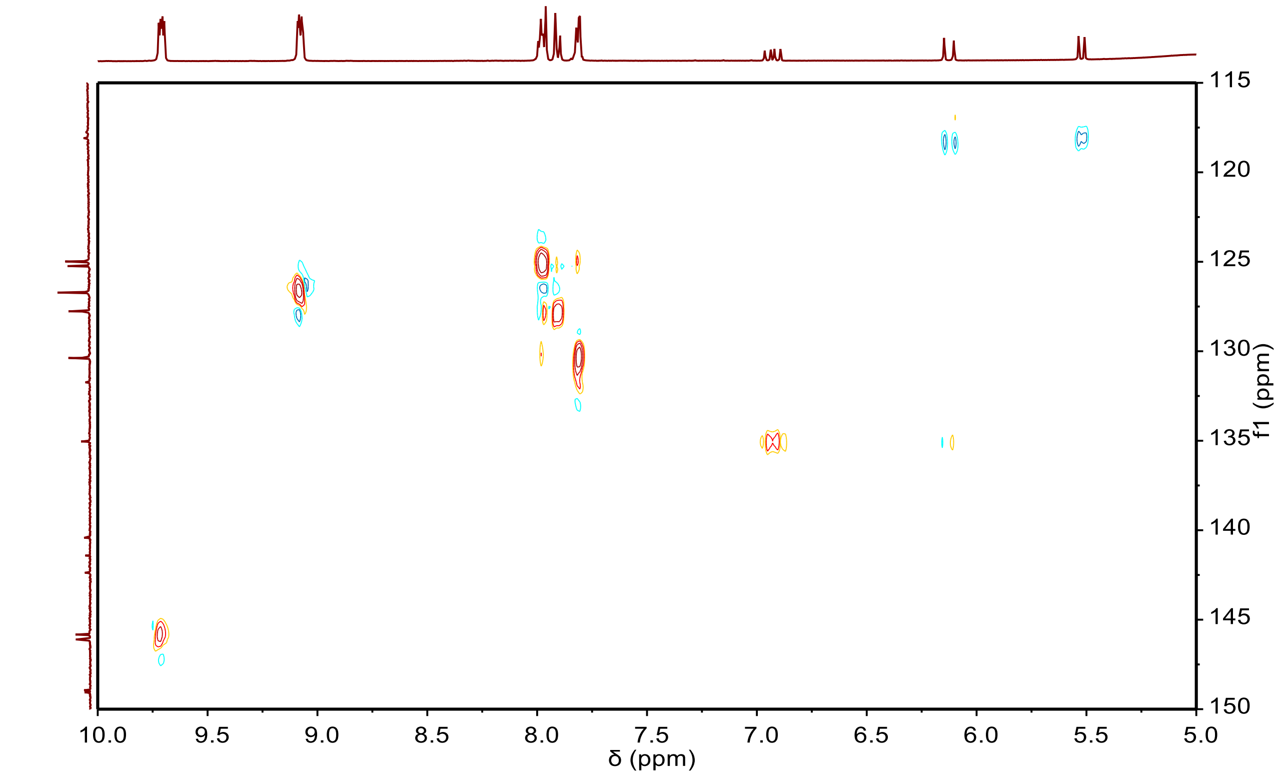

Figure S12. ^{1}H,^{13}C-HSQC NMR spectrum of **$1Cl_2$**.

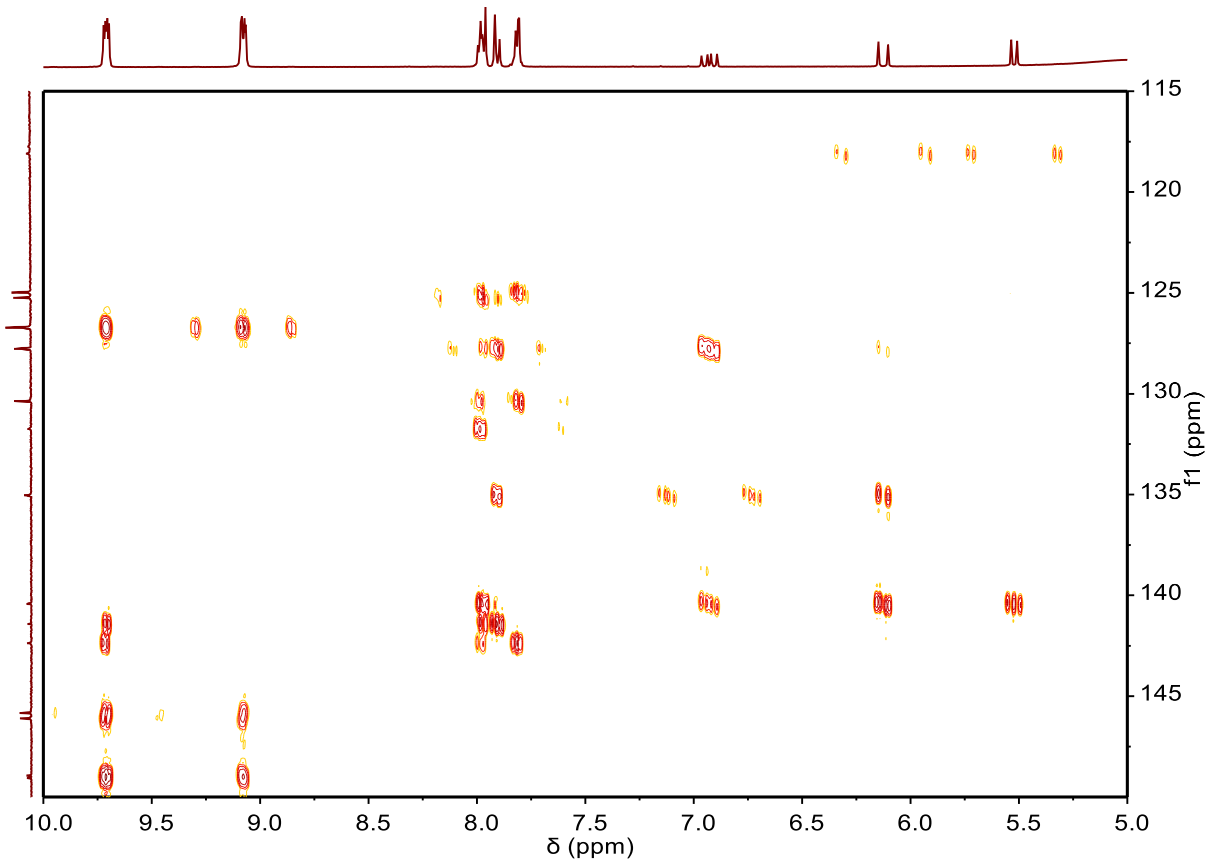

Figure S13. ^{1}H,^{13}C-HMBC NMR spectrum of **$1Cl_2$**.

1.3.6 General procedure for the free radical polymerisation of acrylamide monomers.

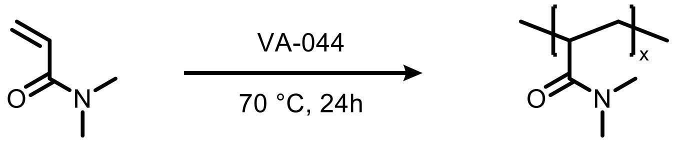

VA-044 (13 μL of a 50 mg/mL solution, 2.02E-3 mmol) was added to a solution of dimethylacrylamide (208 μL, 2.02 mmol) in water (1 mL, [DMAAm] = 2 M) at rt. The reaction mixture was bubbled with Ar for 10-15 min at 0 °C. Next, the reaction mixture was stirred at 70 °C for 24 h to give the product as transparent viscous polymer. M_n = 487,000 g/mol, $Đ$ = 2.32.

Free radical polymerisations with varying parameters were conducted in an analogue manner.

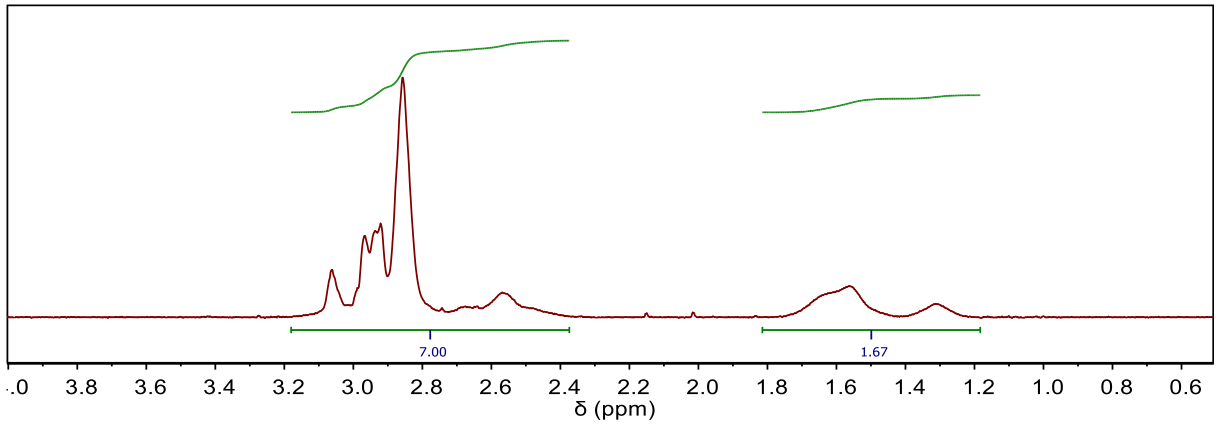

Figure S14. ^{1}H NMR spectrum of poly(DMAAm) in D_2O.

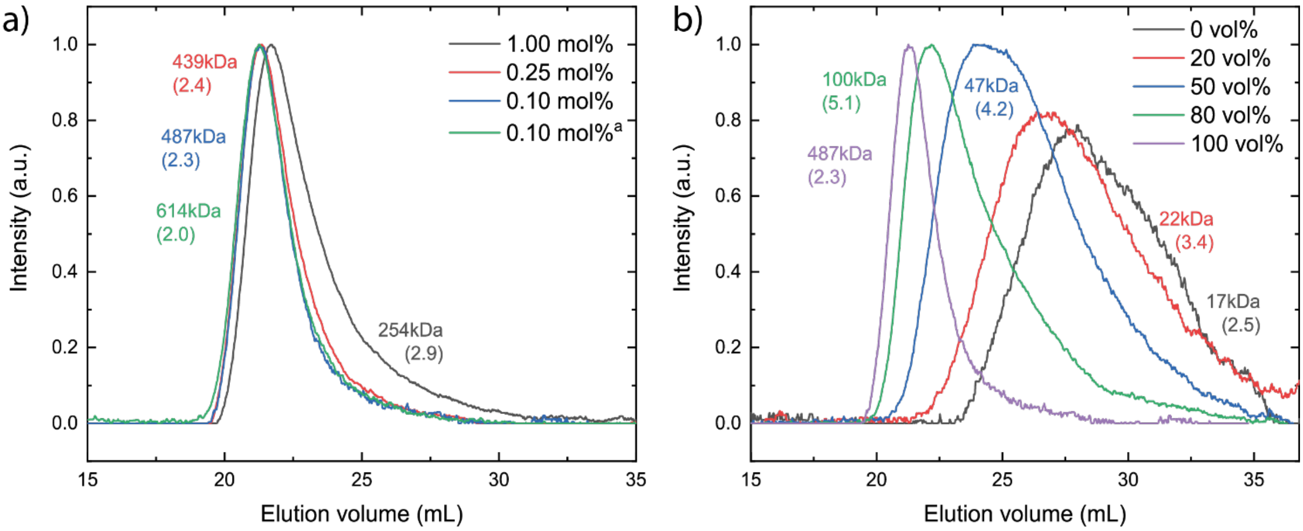

Figure S15. a) SEC traces showing data obtained from refractive index detector of polyDMAAm synthesised in water with varying amounts of initiator. b) SEC traces showing data obtained from refractive index detector of polyDMAAm synthesised with 0.10 mol% initiator and in H_2O-EtOH solvent mixtures with varying vol% of water. The values within the graphs describe the molar masses and dispersity ($Đ$) of the respective polymer SEC trace. [a] This trace was measured for a DMAAm polymerisation after 4 h of reaction. The average molar mass decreases over time (24 h), since the continuous formation initiator radicals over time affords smaller polymer chains that average the overall molar mass to lower values.

Initial polymerisations were carried out in a 97:3 vol% mixture of DMSO and DMF (970/30 μL, [DMAAm] = 2 M) at rt. Conversion was determined via ^{1}H NMR spectroscopy by taking an aliquot of 100 μL after specified times. Various initiators were tested for this polymerisation and the conversion was checked after 15 minutes and 18 h reaction time to assess how fast the polymerisation proceeded (Table S1).

Table S1. Free-radical polymerisations of DMAAm ([M] = 2 M) with varying initiators at 70 °C.

Initiator	mol%	x (15 min)	x (18 h)	M_n (kDa)	*Đ*
AIBN	1.25	82%	98.3%	151	3.07
V-501	1.25	84%	100%	116	3.50
VA-044	1.25	89%	99.0%	134	3.19
VA-044	0.50	92%	99.5%	138	3.60
VA-044	0.10	85%	97.4%	133	3.25
VA-044	0.05	77%	97.6%	193	3.10

Later polymerisations were exclusively conducted in water, as the propagation rate in water is significantly increased due to hydrogen bonding.[5] Analogous polymerisations showed that after 15 min, commonly 100% conversion was obtained.

1.3.7 Synthesis of poly(DMAAm-*stat*-**1**)

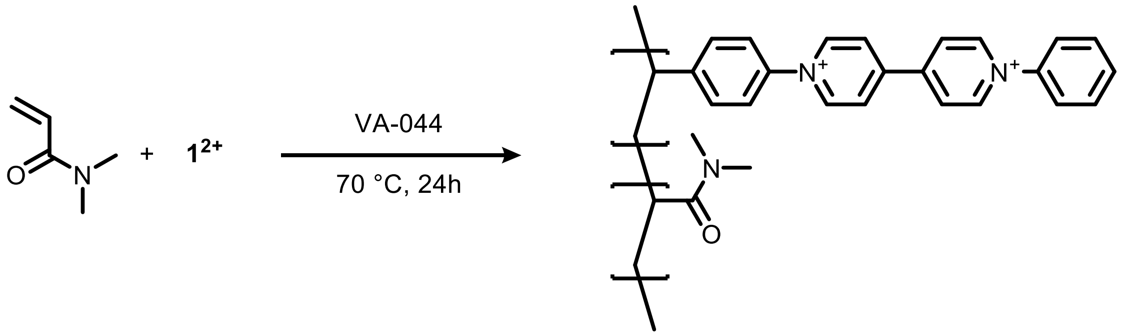

DMAAm (208 μL, 2.02 mmol) and VA-044 (6.53 mg, 2.02E-2 mmol) were added to a solution of **$1Cl_2$** (8.22 mg, 2.02E-2 mmol) in a 97:3 vol% mixture of D_2O and DMF (970/30 μL, [DMAAm] = 2 M) at rt. DMF was added as an internal reference to determine $[DMAAm]_t$ and hence the monomer conversion *via* ^{1}H NMR spectroscopy. The reaction mixture was bubbled with Ar for 10-15 min at 0 °C. Next, the reaction mixture was stirred at 70 °C for 24 h to give the crude product. Purification by dialysis using Spectra/Por dialysis membranes (Spectrumlabs.com, RC, MWCO = 3500 Da) in water overnight prior to freeze-drying gave the product copolymer as slightly yellow viscous liquid. M_n = 27,000 g/mol, *Đ* = 5.30.

Conversion was determined via ^{1}H NMR spectroscopy by taking an aliquot of 100 μL after specified times. Reaction times of 4 h gave conversions of >90%, however the polymerisations were run over

night (~14-18h) to ensure complete conversion. For later experiments we renounced on the use of DMF as internal standard.

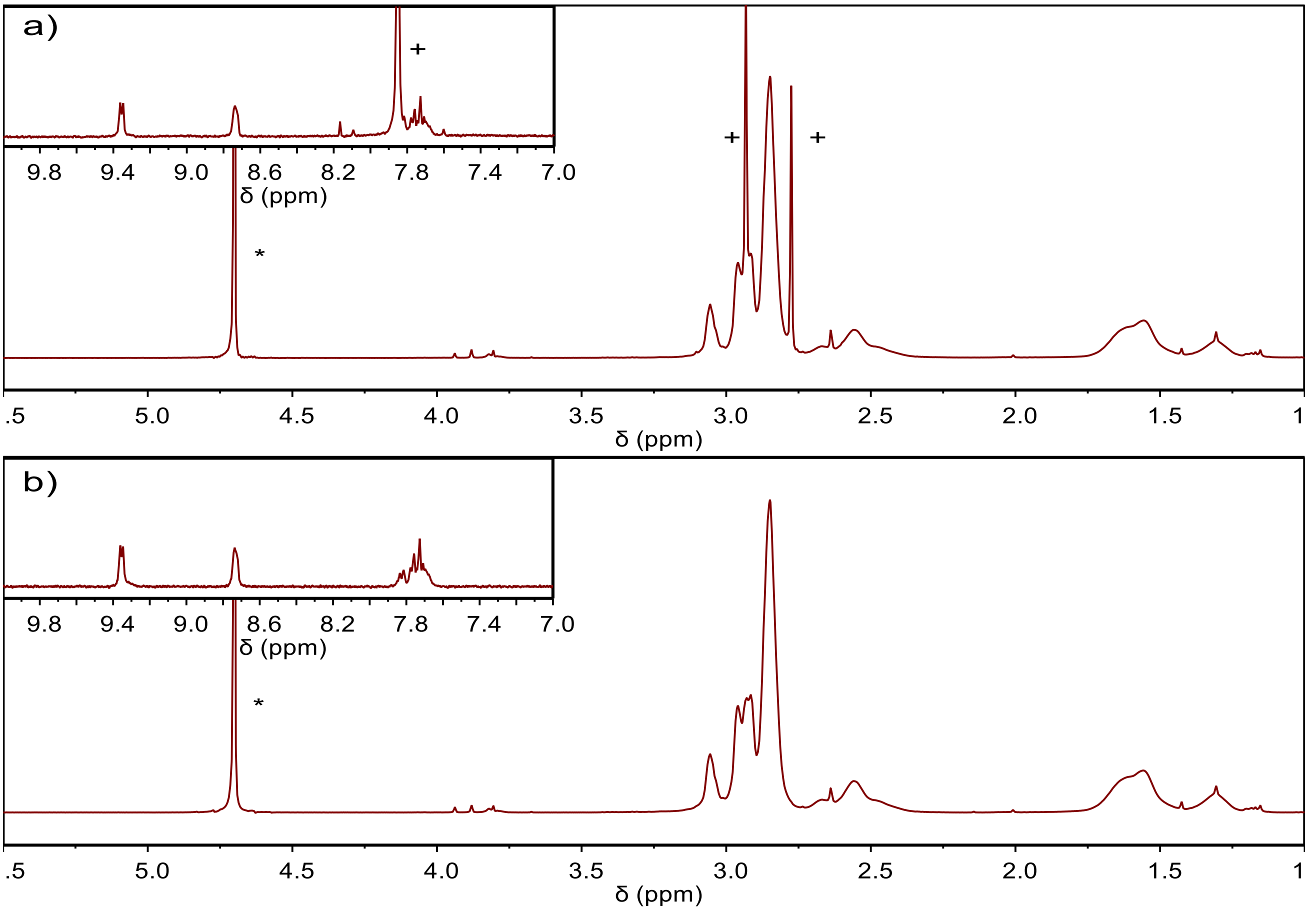

Figure S16. ^{1}H NMR spectrum with inset of crude (a) and dialyzed (b) poly(DMAAm-*stat*-**1**). Residual solvent peaks: D_2O (*), DMF (+). As the vinyl proton signals of $\mathbf{1}^{2+}$ vanish, aryl and bipyridine proton signals appear unshifted but with reduced resolution indicating their incorporation into the main polymer backbone.

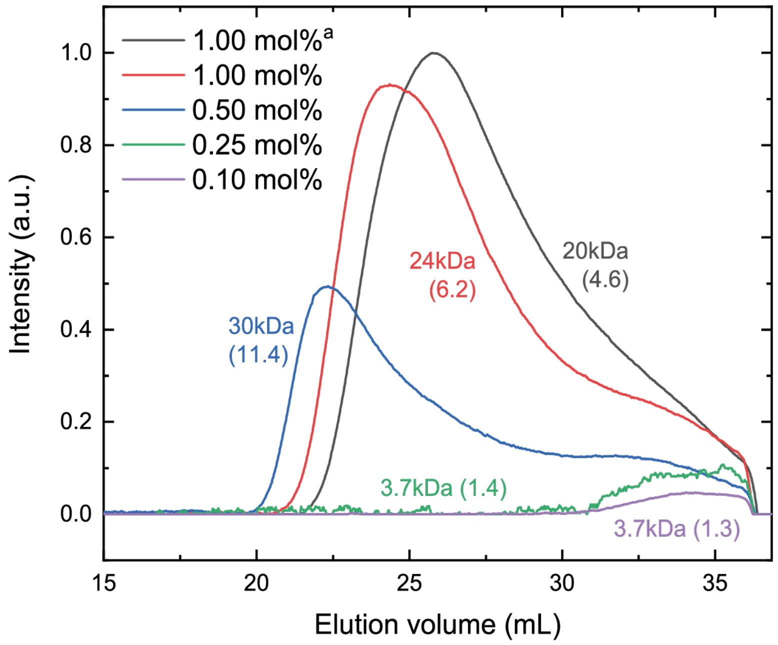

Figure S17. SEC traces showing data obtained from refractive index detector of poly(DMAAm-*stat*-**1**) synthesised in water with 1 mol% of $\mathbf{1}^{2+}$ and varying amounts of initiator. [a] This trace represents DMAAm-co-**1** polymerisation in a 50:50 methanol-water mixture.

1.3.8 General procedure for the CB[*n*]-mediated polymerisation of $\mathbf{1}^{2+}$ and DMAAm

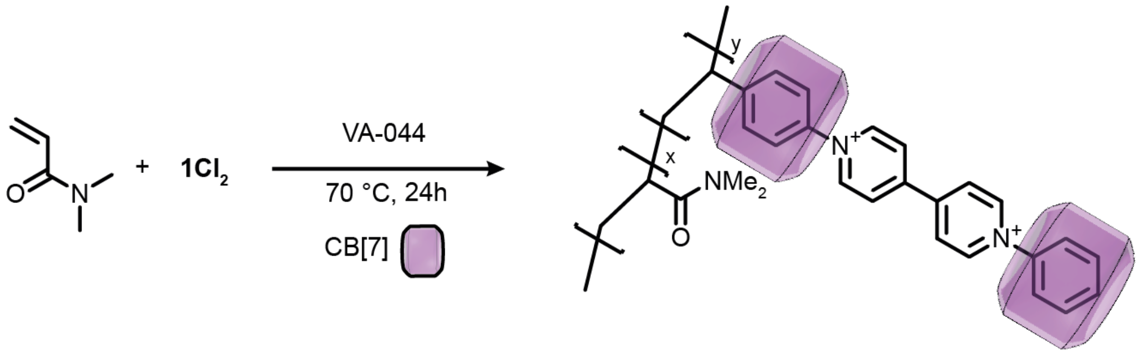

Diarylviologen **1Cl₂** (8.22 mg, 2.02E-2 mmol) and CB[7] (46.9 mg, 4.04E-2 mmol) were dispersed in water (1.00 mL, [DMAAm] = 2 M) at room temperature. The solution was ultrasonicated for 10-15 min to obtain a stable yellow fine dispersion. Then, DMAAm (208 μL, 2.02 mmol) and VA-044 (81.5 μL of a 20 mg/mL solution, 5.05E-3 mmol) were added and the reaction mixture was bubbled with Ar for 10-15 min at 0 °C. Next, the reaction mixture was stirred at 70 °C for 24 h. Then, the solvent was evaporated under reduced pressure and the crude polymer was dissolved in a 0.1 M aqueous solution of 1-adamantylamine. Purification by dialysis using Spectra/Por dialysis membranes (Spectrumlabs.com, RC, MWCO = 3500 Da) in water overnight prior to freeze-drying gave the product copolymer as slightly yellow viscous liquid. M_n = 160,000 g/mol, $Đ$ = 2.43.

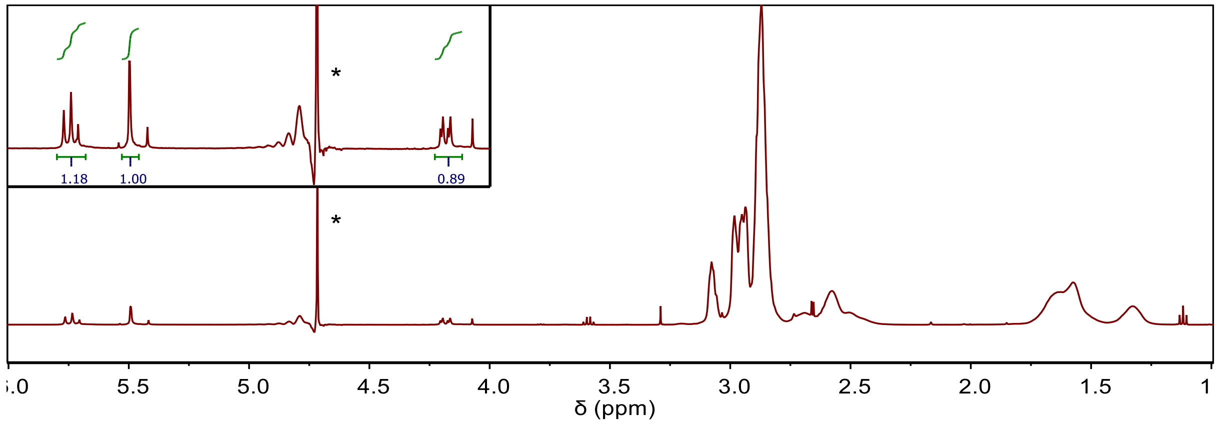

Figure S18. ^{1}H NMR spectrum of supramolecular poly(DMAAm-*stat*-**1**)·CB[7]. Recorded in D_2O (*) with water suppression.

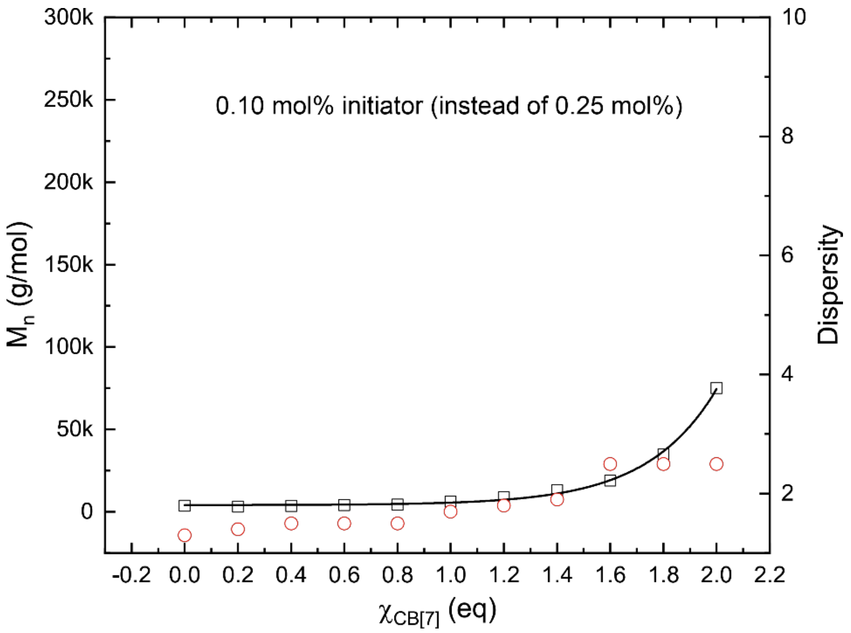

Figure S19. *In situ* copolymerisation of DMAAm ([M] = 2 M) with $\mathbf{1}^{2+}$ (1 mol%) and CB[7] using 0.1 mol% initiator at 70 °C in water. Molar mass (hollow black squares) and dispersity (hollow red spheres) *vs.* amount of CB[7]. Fitted curves is drawn to guide the eye. As opposed to the data obtained with higher initiator concentrations, no maximum in dispersity is observed throughout the varying amount of CB[7] in the system. This is presumably due the very low amounts of initiator used, which are immediately consumed by radical-scavenging monomer $\mathbf{1}^{2+}$ without allowing any polymerisation to happen until the amount of CB[7] reaches higher χ values.

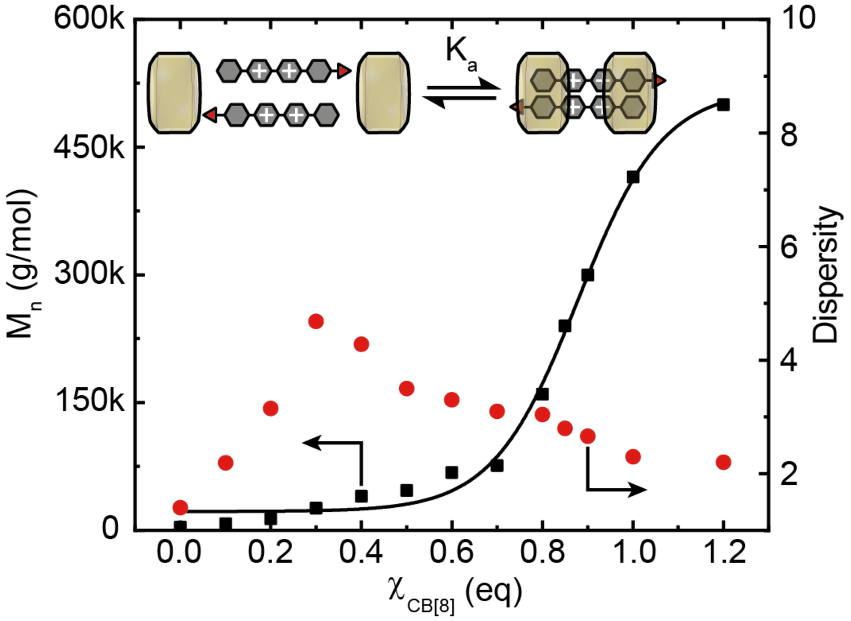

Figure S20. *In situ* copolymerisation of DMAAm with **1**$^{2+}$ and CB[8]. Molar mass (black squares) and dispersity (red spheres) *vs.* amount of CB[8]. Fitted curves are drawn to guide the eye.

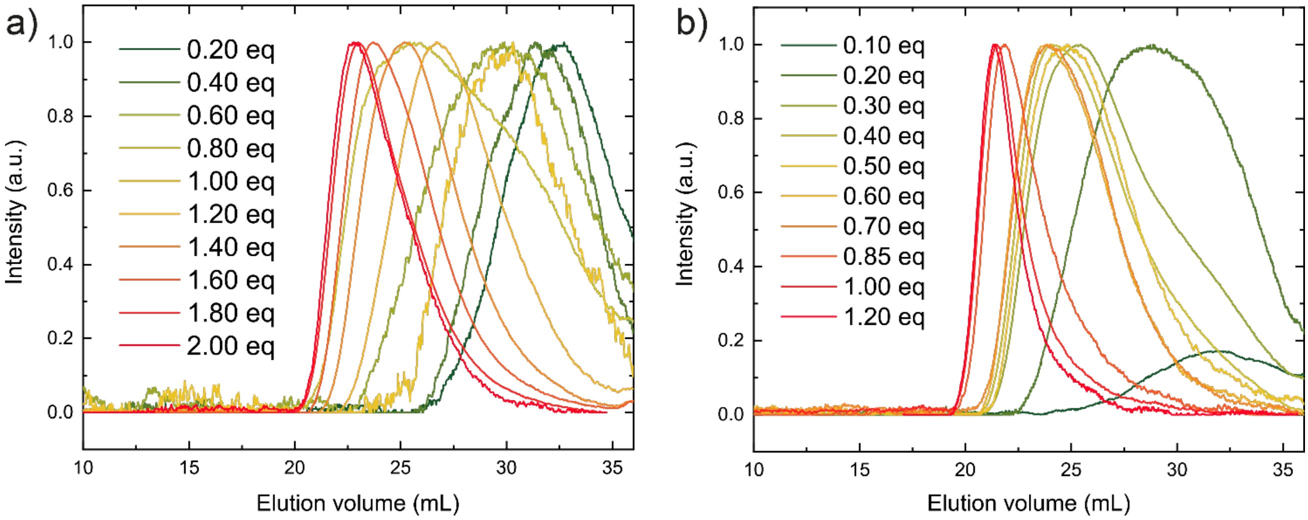

Figure S21. SEC traces showing data obtained from refractive index detector of **1**$^{2+}$-containing copolymers synthesised *via* a) CB[7]- and b) CB[8]-mediated supramolecular protection strategy.

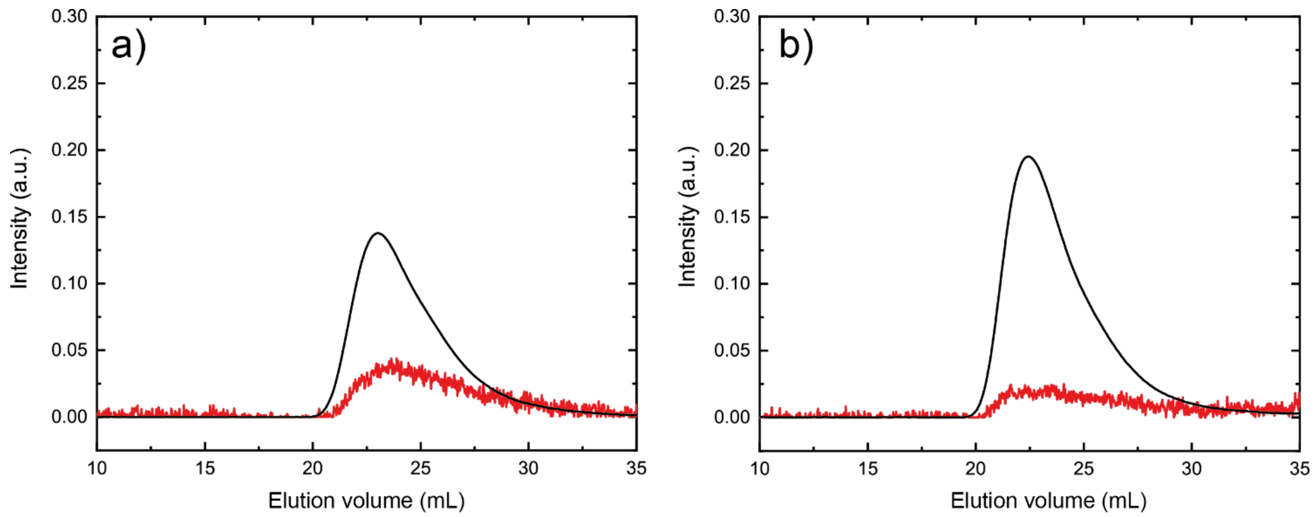

Figure S22. Exemplary SEC elugrams for **1**$^{2+}$-containing DMAAm copolymers synthesised with a) 1.8 mol% CB[7] and b) 0.8 mol% CB[8] showing data obtained from refractive index detector (solid black line) and UV-Vis detector (solid red line).

Removal of CB[8]

For purification of the final copolymers, dialysis with a competitive binder was employed. The crude copolymer product was dissolved in an aqueous solution of 1-adamantylamine (0.1 M). This solution was then dialysed using Spectra/Por dialysis membranes (Spectrumlabs.com, RC, MWCO = 3500 Da) in water overnight prior to freeze-drying to obtain the purified decomplexed copolymer product.

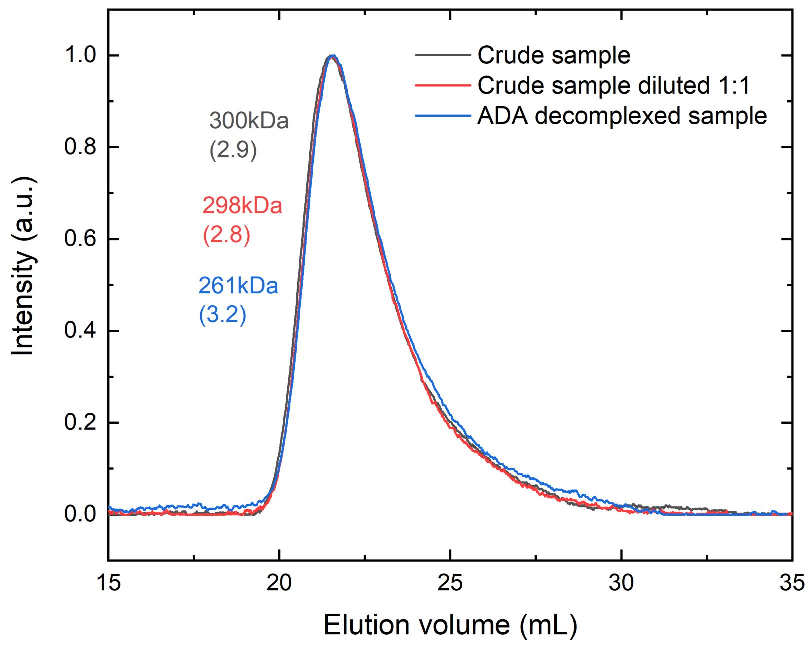

Figure S23. SEC traces showing data obtained from the refractive index detector of different poly(DMAAm-*stat*-**1**) samples. ADA exhibits strong binding affinity towards CB[8] and thus, removes it from the **1**$^{2+}$-containing polymer. The decomplexed sample exhibits reduced molar mass (removal of CB[8]) as compared to the crude sample. This is not a dilution effect, as the 1:1 diluted crude sample exhibits the same molar mass (~300 kDa) as the non-diluted crude sample. Furthermore, the absence of low-molar mass peaks confirms the successful removal of the CB[8]-ADA complex from the purified sample.

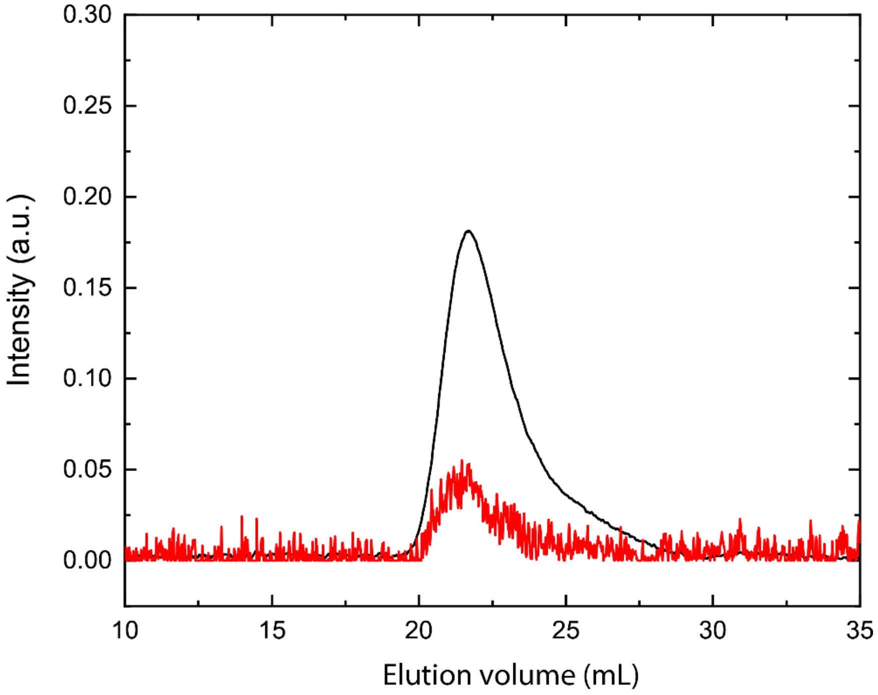

Figure S24. Exemplary SEC elugram for **2**$^{2+}$-containing DMAAm copolymer synthesised with 0.2 mol% of CB[7] showing data obtained from refractive index detector (solid black line) and UV-Vis detector (solid red line).

1.4 Extended discussion on CB[*n*] (*n*=7,8) assisted copolymerisation of DMAAm and **1**$^{2+}$

The suggested mechanism for the free-radical copolymerisation of DMAAm and **1**$^{2+}$ is outlined below. VA-044 acts as the initiator (I) and its thermal activation leads to the formation of two radicals (R•; step **1a**). The first monomer addition between a freshly formed radical and a DMAAm monomer (M) will lead to the formation of a propagating primary radical (RM•; step **1b**). Due to the excess of DMAAm, it can be assumed that this initiation step is predominantly executed *via* addition of a single DMAAm monomer to the radical. Propagation can occur *via* several routes. For example, in step **2a** the primary radical (RM•) can add various monomers M to the growing polymer chain yielding P_n•. In step **2b**, the comonomer **1**$^{2+}$ (instead of a monomer M) can be added to the growing polymer chain to give P_n-**1**$^{2+}$ •. 2c) Once the redox-active monomer **1**$^{2+}$ has been incorporated into the polymer chain, the next monomer is added to the chain (step **2c**). This will likely be another DMAAm monomer due to its large excess. Termination may occur *via* combination of two radical-containing polymer chains (step **3a**) or disproportionation (step **3b**). Beside engaging in the polymerisation, monomer **1**$^{2+}$ may react with radicals on its own to cause a retardation of the overall polymerisation (step **4a** and step **4b**). As such, monomer **1**$^{2+}$ may react with the freshly formed radical R• to form R (inactive), radical cation **1**$^{+\bullet}$ and a proton H^+ *via* oxidative termination. Analogously, a radical of a growing polymer chain P_m• may be transferred to **1**$^{2+}$ via this pathway delivering a deactivated polymer chain (P_m=), radical

cation $\mathbf{1}^{+\bullet}$ and a proton H^+. Such "oxidative terminations" are commonly observed in the aqueous polymerisations of methyl methacrylate, styrenes and also acrylonitriles using redox initiator systems.[6-9] Similar observations have been reported when methylene blue was used as a retarder, where the primary radical is transferred to a methylene blue electron acceptor *via* oxidative termination.[10] This transfer may equally occur from initiating species such as radical azo initiators (as in step 4a).[11]

Initiation			
1a)	I (VA-044)	⟶	2 R•
1b)	R• + M	⟶	RM•
Propagation			
2a)	RM• + M	⟶	P_n•
2b)	P_n• + $\mathbf{1}^{2+}$	⟶	P_n-$\mathbf{1}^{2+}$•
2c)	P_n-$\mathbf{1}^{2+}$• +M	⟶	P_n-$\mathbf{1}^{2}$-M•
Termination			
3a)	P_n• + P_m•	⟶	P_n-P_m
3b)	P_n• + P_m•	⟶	P_n-H + P_m=
Retardation			
4a)	R• + $\mathbf{1}^{2+}$	⟶	R + $\mathbf{1}^{+\bullet}$ + H^+
4b)	P_m• + $\mathbf{1}^{2+}$	⟶	P_m= + $\mathbf{1}^{+\bullet}$ + H^+
Protection			
5a)	P_m• + $\mathbf{1}$·$CB[7]_2$	⟶ (crossed)	P_m= + $\mathbf{1}$·$CB[7]_2$ + H^+
5b)	P_m• + $(\mathbf{1})_2$·$CB[8]_2$	⟶ (crossed)	P_m= + $(\mathbf{1})_2$·$CB[8]_2$ + H^+

The retardation process may be supressed when monomer $\mathbf{1}^{2+}$ is encapsulated within CB[n] macrocycles (step **5a** and step **5b**). Upon addition of CB[7] and CB[8], the formation of $\mathbf{1}^{2+}$•$(CB[7])_2$ and $(\mathbf{1}^{2+})_2$•$(CB[8])_2$ complexes result in shielding of the redox-active part of monomer $\mathbf{1}^{2+}$ from other radical species within the system (*i.e.* R•, RM•, P_n• *etc.*), hampering side electron transfer processes. This results in polymerisation proceeding with minimal disruption, mitigating the risk of radical transfer from the propagating polymer chain to the viologen electron acceptors. Moreover, by inhibiting the formation of $\mathbf{1}^{+\bullet}$ through supramolecular encapsulation, the probability of forming either π- or σ- dimers is decreased further preventing the reaction system from any additional processes that could affect the overall molecular weight or polydispersity of the resulting polymers.

1.5 NMR binding studies

Solutions of the guest molecule are titrated to solutions of CB[*n*] (n=7 or 8) in D_2O until the discrete binding modes are observed. CB[*n*] macrocycles arrange around the long-axis of the guest molecules either forming a 1:2 complex with CB[7] or a 2:2 complex with CB[8]. The spatial position of the macrocycles around the guest molecules changes the chemical environment. As a consequence, ^{1}H

NMR signals of protons located in the CB[*n*] cavity experience upfield shifts (up to 1 ppm), whereas ^{1}H NMR signals of protons located outside or close to the carbonyl-laced CB[*n*] portals display downfield shifts.[12] CB[7] isolates a discrete monomer of $\mathbf{1}^{2+}$ by forming $\mathbf{1}\cdot CB[7]_2$ (Figure S25).

While the signals of the aromatic bipyridine protons experience only little shifts, the aryl proton signals shift upfield by δ = 0.557 - 1.00 ppm depending on the location of the CB macrocycle on this residue. Two sets of doublets are clearly visible at δ = 7.90 - 7.75 ppm, which correspond to the *ortho* and *meta*-protons of the styryl unit. The triplet at δ = 7.25 ppm is assigned to the single proton in *para*-position of the phenyl substituent. Both signals of the *ortho* and *meta*-phenyl protons overlap in the region around δ = 7.00 – 6.85 ppm (triplet and doublet). The vinyl C*H* proton, originally a doublet of doublets situated at δ = 6.93 ppm, shifts upfield to δ = 6.27 ppm upon encapsulation with CB[7]. The vicinal *trans*- and *cis*-proton signals (δ = 6.06 and 5.54 ppm, respectively), display similar shifts and relocate at δ = 5.58 and 5.27 ppm, respectively. These findings suggest that each aryl residue is shielded by one CB[7] macrocycle. The diffusion constant of $\mathbf{1}\cdot CB[7]_2$ is reduced to D = 2.35 x 10^{-10} m^2s^{-1} as compared to free $\mathbf{1}^{2+}$ (D = 4.92 x 10^{-10} m^2s^{-1}). CB[8]-mediated complexation of $\mathbf{1}^{2+}$ delivers a dimeric stack. The general signal shifts of $\mathbf{1}^{2+}$ upon complexation with CB[8] stay in the range of δ = 0.56 - 1.17 ppm and δ = 0.72 - 0.77 ppm for aryl and vinyl proton signals, respectively (Figure S26). Additionally, the formation of two different isomers is witnessed by the signal splitting of protons in *para*-position of the bipyridine substituents. The h2h orientation is less favored as both slightly bulkier vinyl groups are situated on the same site and thus experience repulsion. In attaining the h2t conformation, a centrosymmetric geometry with an inversion center and both vinyl groups facing apart is formed. Integration of these signals reveals a 60:40 ratio of the h2t to h2h conformation of the host-guest complex. The imbalance towards the h2t stereoisomer is expected, as this conformation is sterically less demanding. If the amount of guest in the system is increased, proton signals of the complex become broad and reduced in intensity; simultaneously, broad proton signals of the free monomer $\mathbf{1}^{2+}$ appear. This effect is symptomatic for an increasingly dynamic complexation, where complexed guest molecules exchange with free solvated guest molecules on a regular time scale. Further and as expected, the diffusion constant of $(\mathbf{1})_2\cdot CB[8]_2$ decreases (D = 2.25 x 10^{-10} m^2s^{-1}) as compared to $\mathbf{1}\cdot CB[7]_2$ and free monomer $\mathbf{1}^{2+}$.

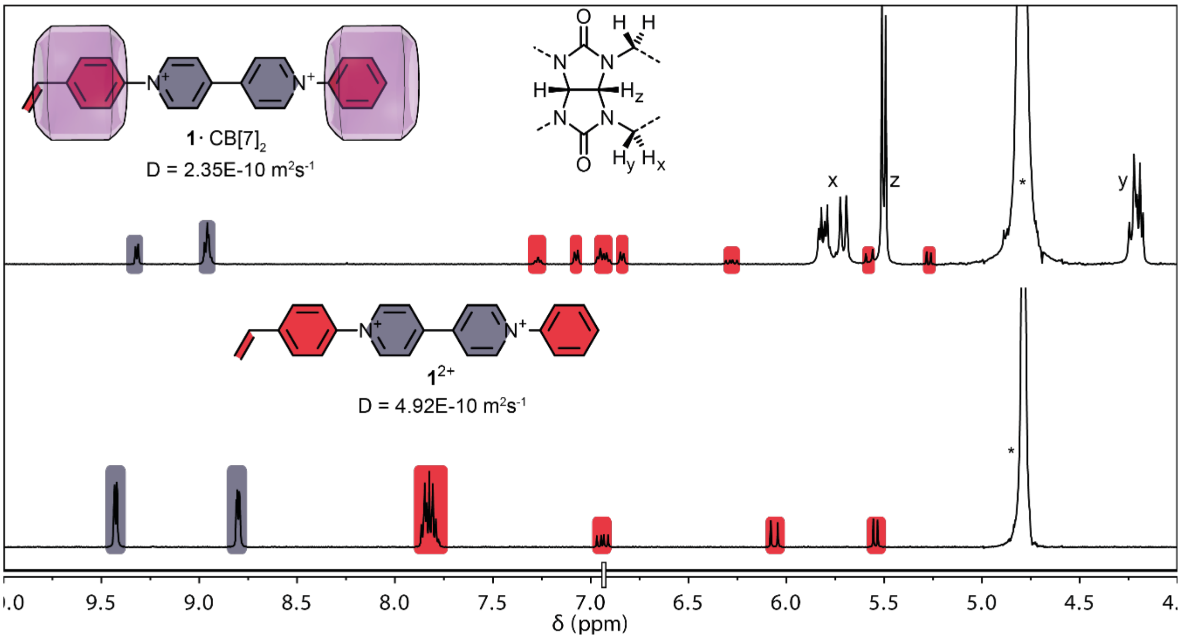

Figure S25. ^{1}H NMR spectra (500 MHz) of **1**$^{2+}$ (bottom) and its complexation **1**·CB[7]$_2$ (top) in D_2O (*) at 298 K.

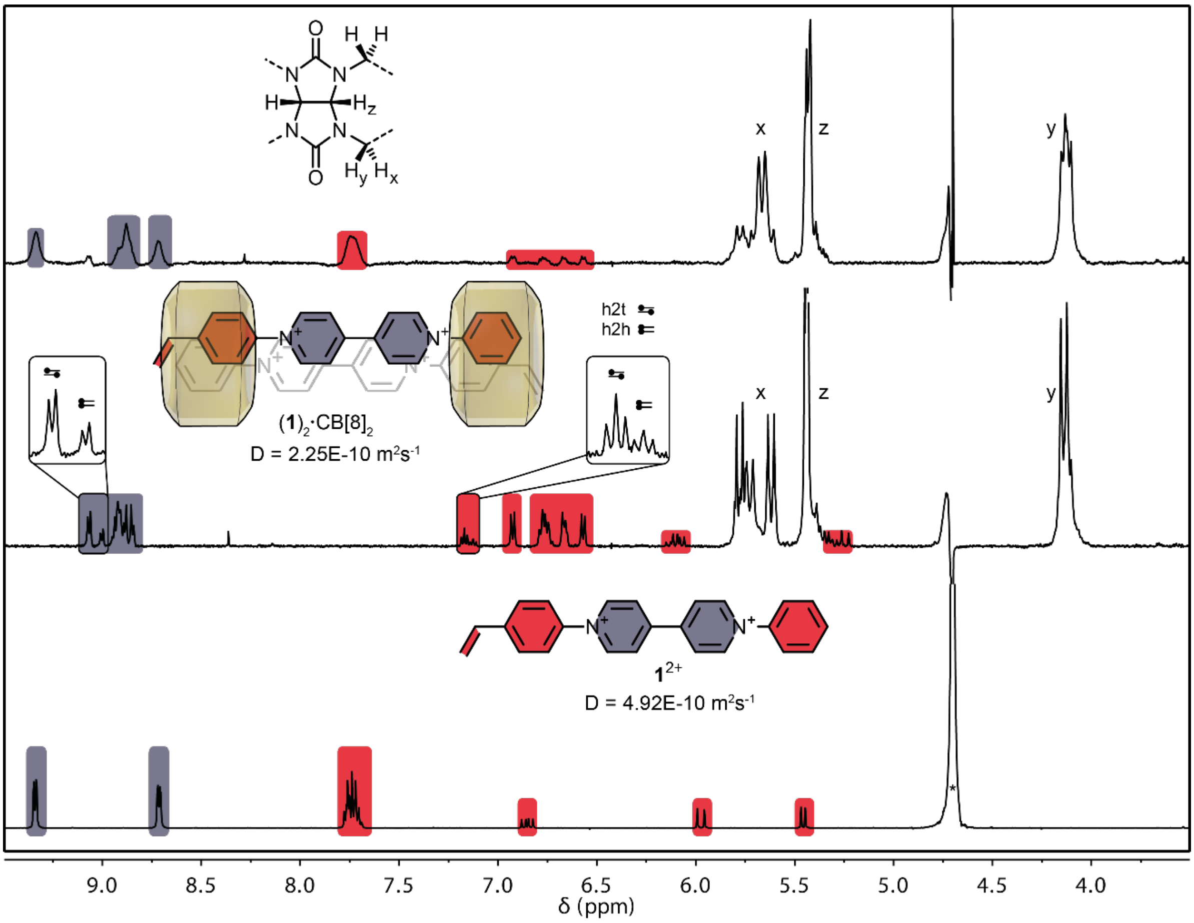

Figure S26. ^{1}H NMR spectra (500 MHz) of **1**$^{2+}$ (bottom) and its complexation (**1**)$_2$·CB[8]$_2$ (middle) in D_2O (*) at 298 K. Two guest molecules of **1**$^{2+}$ in CB[8] can assume a head-to-head (h2h) or a head-to-tail (h2t) conformation (inset graphs, middle spectrum). The h2t conformation is slightly preferred and makes up 60% of the complex species. The top spectrum shows a transition from the 2:2 complex towards a more dynamic complexation equilibrium.

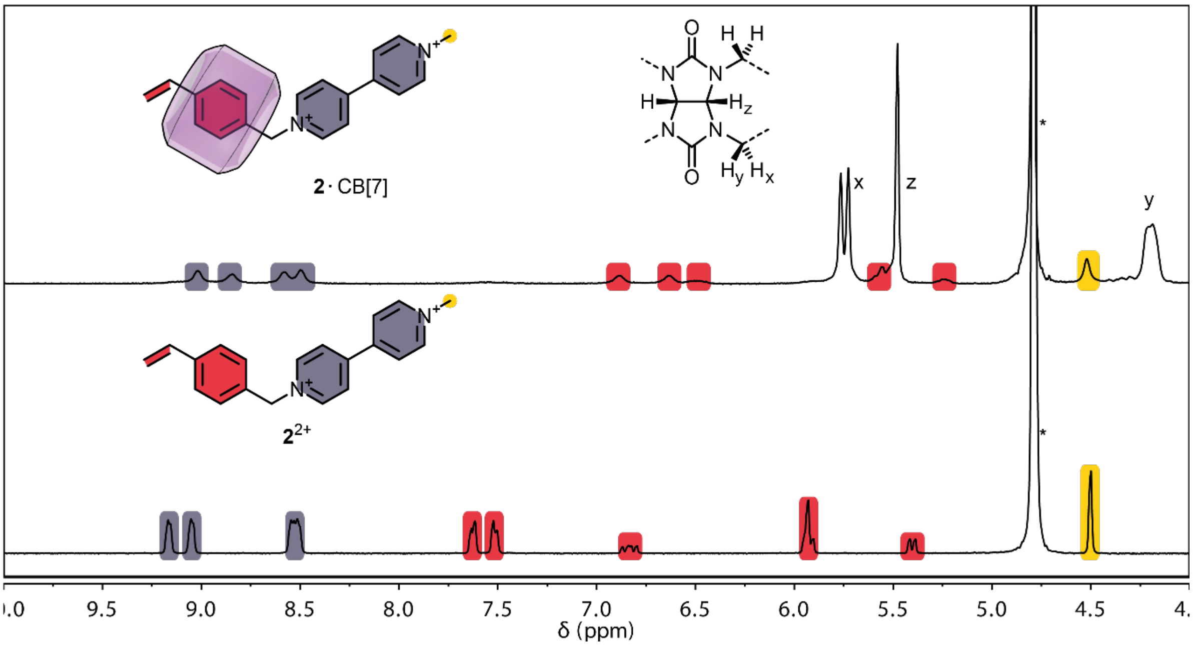

Figure S27. ^{1}H NMR spectra (500 MHz) of **2**$^{2+}$ (bottom) and its complexation **2**·CB[7] (top) in D_2O (*) at 298 K.

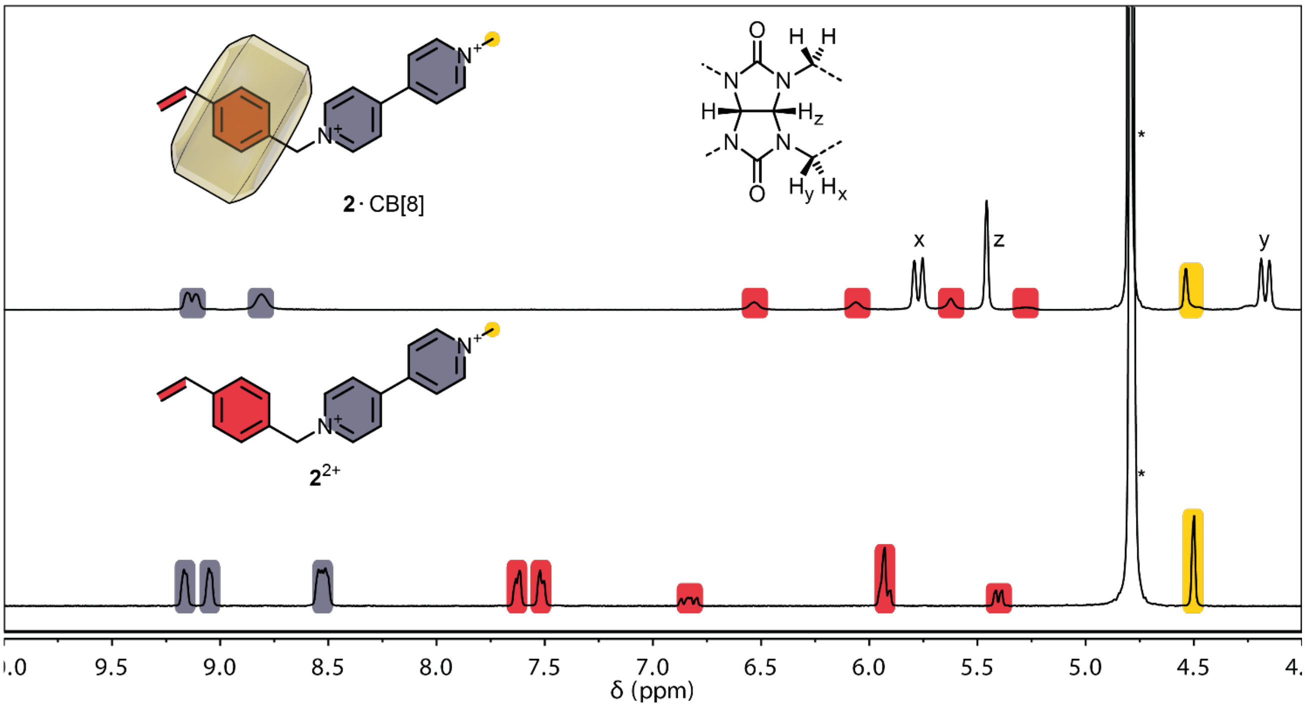

Figure S28. ^{1}H NMR spectra (500 MHz) of **2**$^{2+}$ (bottom) and its complexation **2**·CB[8] (top) in D_2O (*) at 298 K.

1.6 ITC data

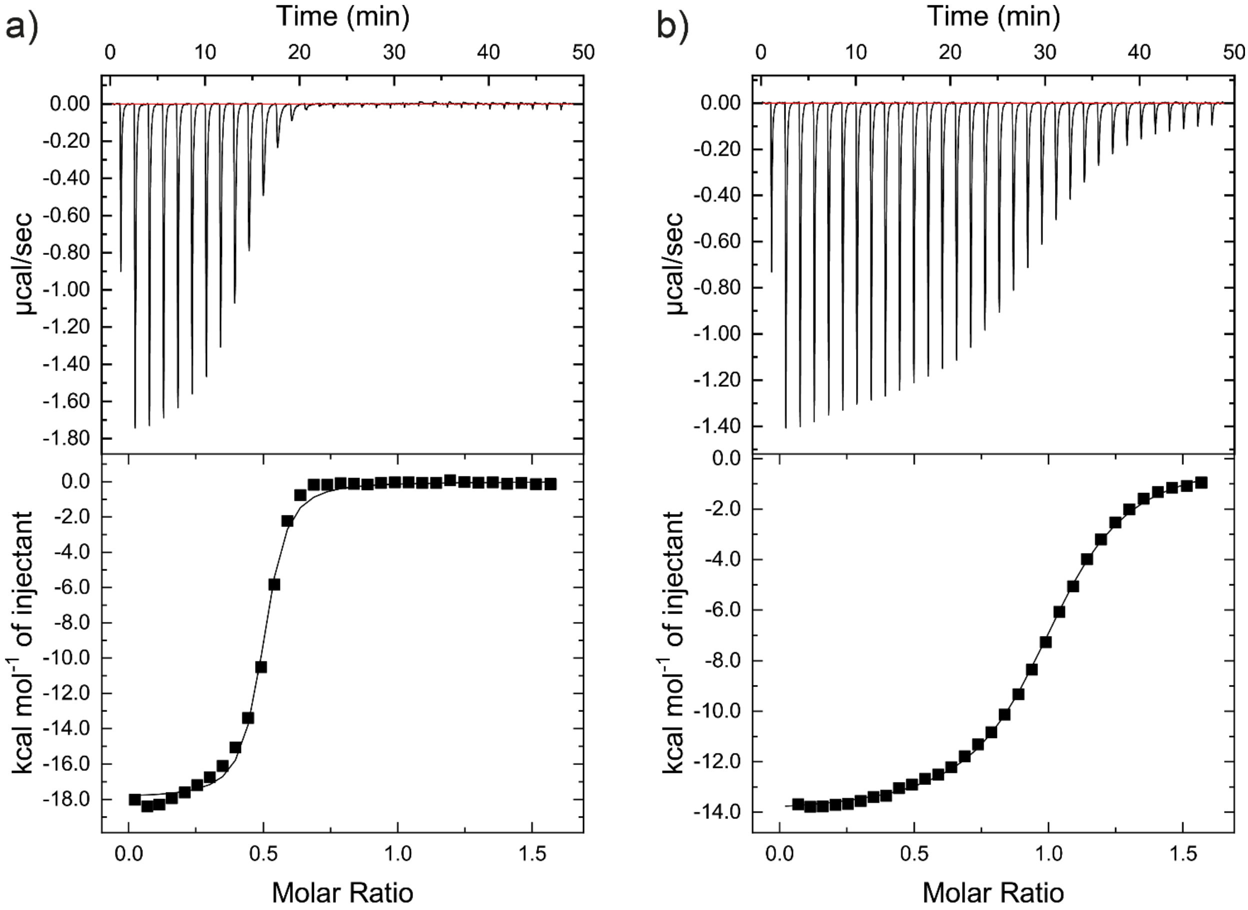

Figure S29. ITC titration curves obtained by titrating a) CB[7] (0.1 mM) and b) CB[8] (0.1 mM) to an aqueous solution of **1**$^{2+}$ (0.747 mM).

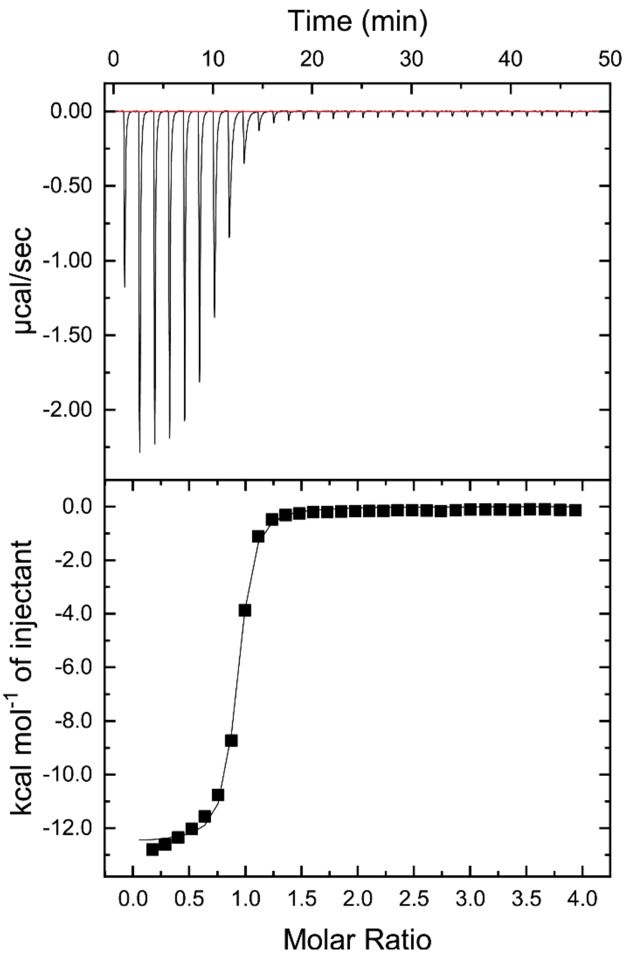

Figure S30. ITC titration curves obtained by titrating CB[7] (0.1 mM) to an aqueous solution of $\mathbf{2}^{2+}$ (1 mM).

Table S2. Overview of the thermodynamic data for the CB[*n*] complexation of different guest molecules by ITC in pure H_2O at 298.15 K.

	**1**·CB[7]$_2$	(**1**)$_2$·CB[8]$_2$	**2**·CB[7]
K [M^{-1}]	2.64E6 ± 3.18E5	9.02E10 ± 1.37E8	2.32E6 ± 2.27E5
ΔH [cal/mol]	-1.793E4 ± 157.4	-28.14E4	-1.252E4 ± 87.63
ΔS [cal/mol/K]	-30.8	-42.6	-12.9
TΔS [cal/mol]	-9.183E3	-12.7E3	-3.844E3
ΔG [cal/mol]	-8.747E3	-15.44E3	-8.676E3

1.7 Cyclic Voltammetry

As a general procedure, a 1 mM stock solution of monomer **1**$^{2+}$ was prepared in 0.1 $NaCl_{(aq)}$. The stock solution was split in aliquots of 4 mL and respective equivalents of CB[*n*] were added prior to sonication of each individual sample solution. Sample solutions were then transferred to the electrochemical cell and bubbled with N_2 for 10 min prior to starting the CV measurement.

Initial experiments focused on the complexation of **1**$^{2+}$ with excess of CB[*n*] (*n*= 7 or 8) (Figure S28).

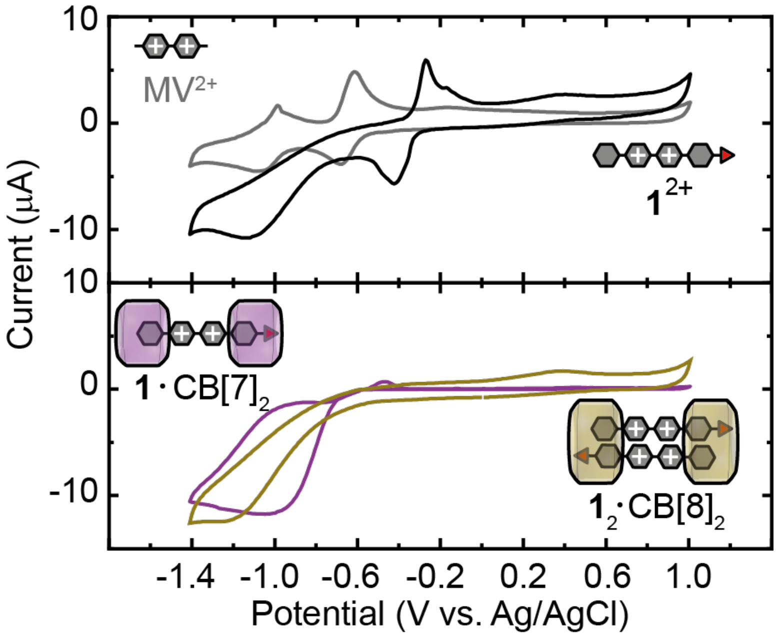

Figure S31. Cyclic voltammetry of methyl viologen (MV^{2+}) and **1**$^{2+}$ (1 mM in 0.1 M NaCl, top) and in the presence of excess CB[*n*] macrocycles (bottom) at a scan rate of 10 mV/s in 0.1 N $NaCl_{(aq)}$ at 25 °C.

Compound **1**$^{2+}$ shows a quasi-reversible reduction wave at -0.44 V, where the radical-cation **1**$^{+\bullet}$ is formed. A second cathodic peak is represented by an irreversible reduction wave at -1.13 V, where the neutral and water-insoluble species **1**^{0} is generated. Upon reaching the switching potential (- 1.4 V), the scan proceeds in anodic direction passing the major anodic peak at -0.27 V, which is assigned to the reoxidation of **1**$^{+\bullet}$ to **1**$^{2+}$. Upon measuring multiple cycles, cathodic peaks experience fading accompanied by a minor shift towards more negative potentials, due to electrode adsorption phenomena (Figure S29a). Methyl viologen shows two reversible reduction waves at -0.70 and -1.01 V (Figure S28, top). Comparatively, both half-wave potentials of **1**$^{2+}$ are shifted towards more positive potentials, on account of the electron-withdrawing nature of the aryl modifications and thus the molecule's improved ability to stabilise electrons.[13] Using excess CB[7] (to form **1**•$CB[7]_2$), a cathodic peak is observed at -0.47 V, strongly reduced in intensity (Figure S28, bottom). The addition of excess CB[8] to form $(\mathbf{1})_2$•$CB[8]_2$, successfully shields **1**$^{2+}$ from taking part in electron transfer

processes. Periodic cycling of the exerted potential reveals that both self-assemblies remain stable and inaccessible to electrons generated (Figure S29b and c).

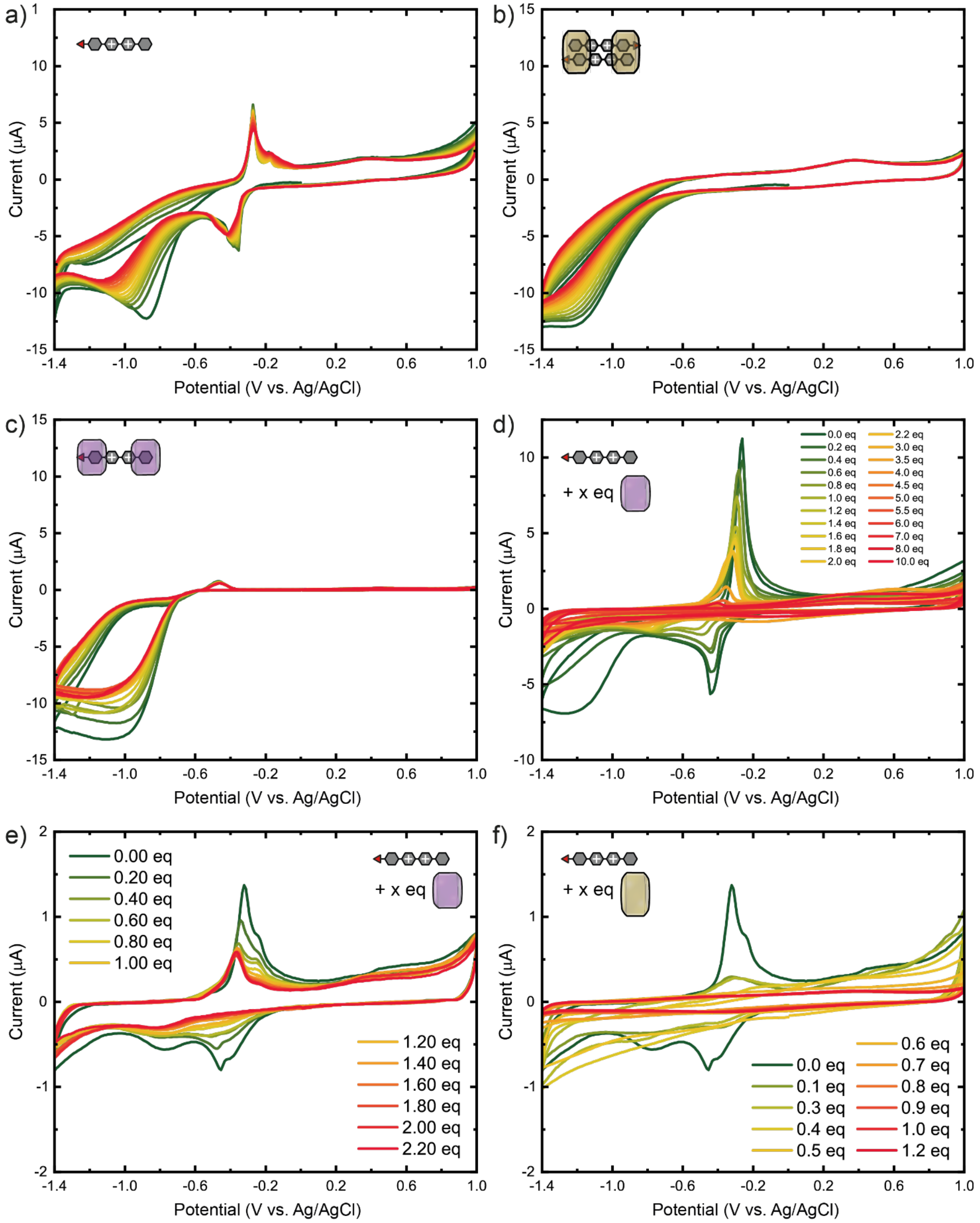

Figure S32. Cyclic voltammetry of a) $\mathbf{1}^{2+}$ (10 mV/s, 15 scans), b) $(\mathbf{1})_2$·CB[8]$_2$ (excess CB[8], 10 mV/s, 15 scans), c) **1**·CB[7]$_2$ (excess CB[7], 10 mV/s, 15 scans), d) $\mathbf{1}^{2+}$ + x equiv. CB[7] (100 mV/s), e) $\mathbf{1}^{2+}$ + x equiv. CB[7] (10 mV/s) and f) $\mathbf{1}^{2+}$ + x equiv. CB[8] (10 mV/s). Analyte concentration was maintained at 1 mM in 0.1 mM $NaCl_{(aq)}$ solution.

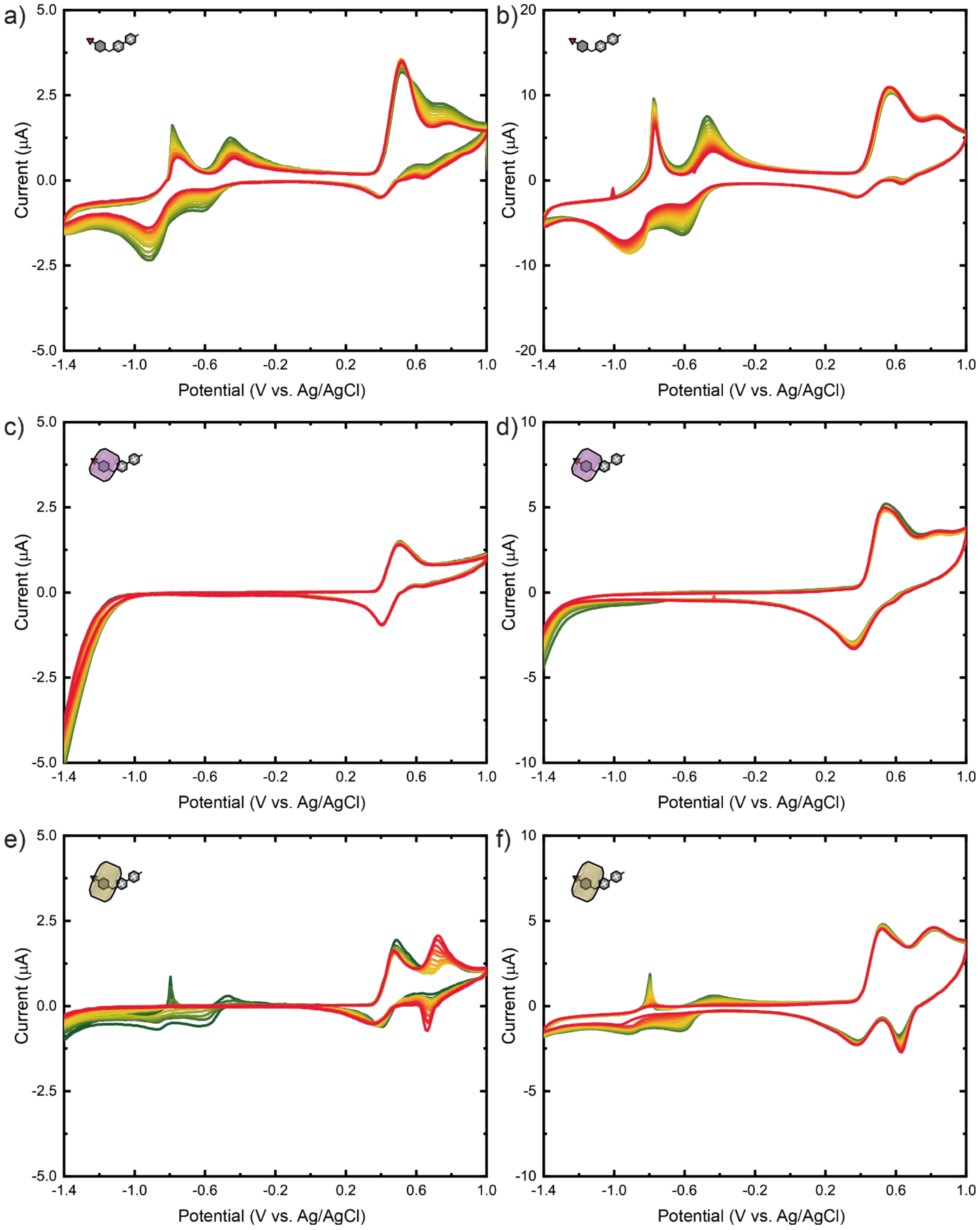

Figure S33. Cyclic voltammetry of a) $\mathbf{2}^{2+}$ (10 mV/s), b) $\mathbf{2}^{2+}$ (100 mV/s), c) $(\mathbf{2})_1$·CB[7]$_1$ (excess CB[7], 10 mV/s), d) **2**·CB[7] (excess CB[7], 100 mV/s), e) **2**·CB[8] (excess CB[8], 10 mV/s) and f) **2**·CB[8] (excess CB[8], 100 mV/s). Analyte concentration was maintained at 1 mM in 0.1 mM $NaCl_{(aq)}$ solution. Each measurement was subjected to 15 consecutive scans.

1.8 UV-Vis Experiments

UV-Vis spectra of the corresponding samples with varying concentration of CB[*n*] were prepared as follows: Aliquots (2 mL) of each previously measured CV sample were separated in a 14 mL Falcon tube and bubbled with Ar for 10 min. Next, sodium dithionite (20 equiv.) was added towards the deoxygenated solutions to yield a mixture of $\mathbf{1}^{2+}$ and reduced species $\mathbf{1}^{\bullet+}$ and $\mathbf{1}^{0}$. In case of an analyte concentration of 1 mM, precipitate occurred, which was separated by centrifugation under Ar at 10,000 rpm for 10 min. The remaining supernatant was then transferred to an Ar-purged UV-Vis cuvette prior to its measurement on the UV-Vis spectrometer. For samples with analyte concentrations of 0.1 mM, no precipitate was formed during the reduction step and hence, the centrifugation step was omitted for these samples.

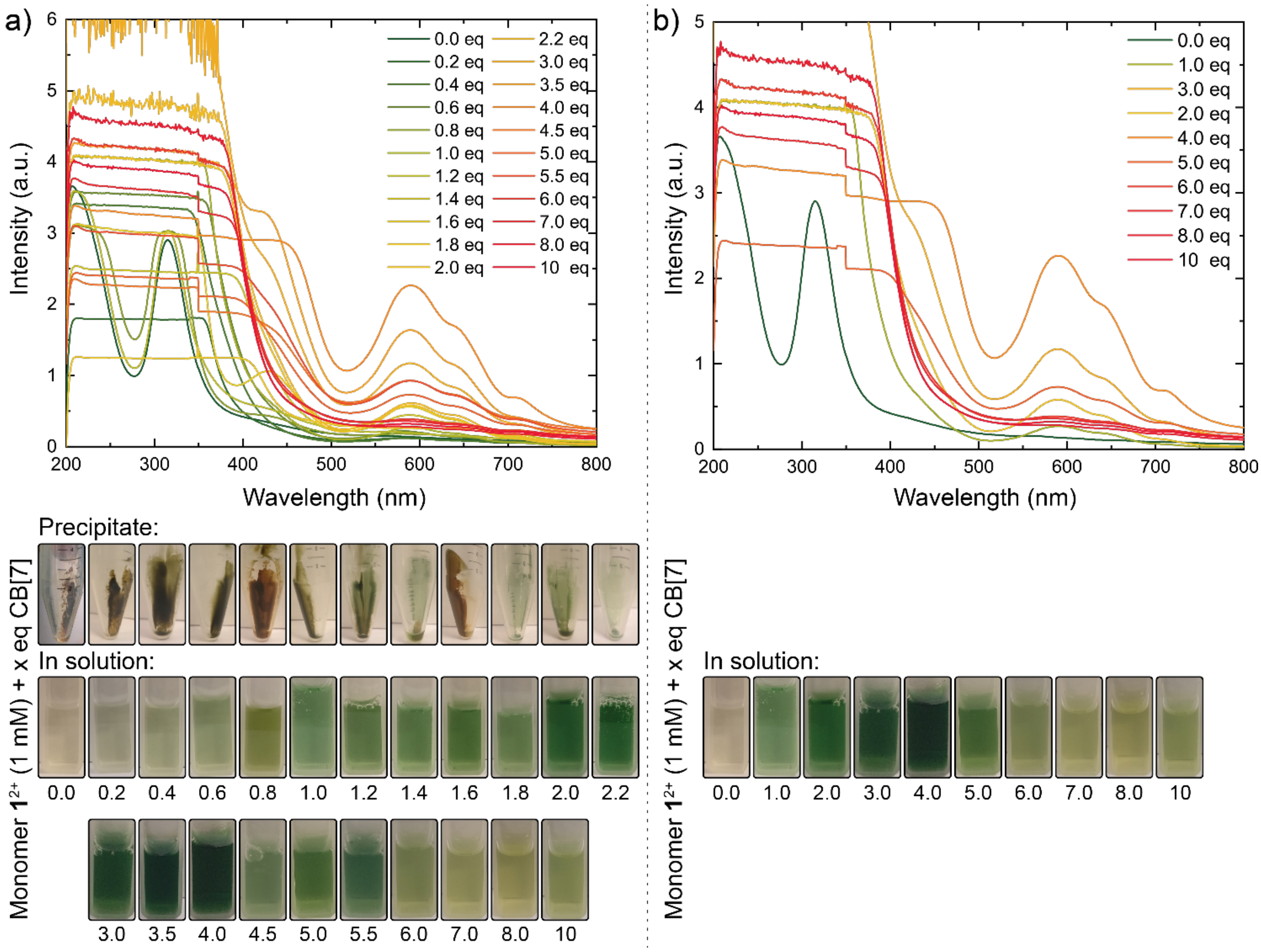

Figure S34. a) Complete UV-Vis dataset (top) of compound $\mathbf{1}^{2+}$ (1 mM) with individual amounts of CB[7] and sample photographs (bottom). b) Selected UV-Vis spectra (top) of compound $\mathbf{1}^{2+}$ (1 mM) with individual amounts of CB[7] and corresponding sample photographs (bottom).

2 References

1. Brautigam, C. A.; Zhao, H.; Vargas, C.; Keller, S.; Schuck, P., Integration and global analysis of isothermal titration calorimetry data for studying macromolecular interactions. *Nature Protocols* **2016,** *11* (5), 882-894.
2. Yamaguchi, I.; Higashi, H.; Shigesue, S.; Shingai, S.; Sato, M., N-Arylated pyridinium salts having reactive groups. *Tetrahedron Lett.* **2007,** *48* (44), 7778-7781.
3. Nimkar, S. K.; Anderson, A. H.; Rimoldi, J. M.; Stanton, M.; Castagnoli, K. P.; Mabic, S.; Wang, Y. X.; Castagnoli, N., Synthesis and Monoamine Oxidase B Catalyzed Oxidation of C-4 Heteroaromatic Substituted 1,2,3,6-Tetrahydropyridine Derivatives. *Chemical Research in Toxicology* **1996,** *9* (6), 1013-1022.
4. Zhang, K.-D.; Tian, J.; Hanifi, D.; Zhang, Y.; Sue, A. C.-H.; Zhou, T.-Y.; Zhang, L.; Zhao, X.; Liu, Y.; Li, Z.-T., Toward a Single-Layer Two-Dimensional Honeycomb Supramolecular Organic Framework in Water. *Journal of the American Chemical Society* **2013,** *135* (47), 17913-17918.
5. Valdebenito, A.; Encinas, M. V., Effect of solvent on the free radical polymerisation of N,N-dimethylacrylamide. *Polym. Int.* **2010,** *59* (9), 1246-1251.
6. Fernandez, M. D.; Guzman, G. M., Cellobiose-Ce(IV) as a redox initiating system for aqueous polymerisation of methyl methacrylate. *Eur. Polym. J.* **1990,** *26* (3), 301-307.
7. Billaud, C.; Sarakha, M.; Bolte, M., Kinetic study of azidopentaammine cobalt(III) complex as photoinitiator and termination agent of N-vinylpyrrolidone radical polymerisation. *J. Polym. Sci., Part A: Polym. Chem.* **2000,** *38* (21), 3997-4005.
8. Fernández, M. D.; Guzmán, G. M., Aqueous polymerisation of methyl methacrylate initiated by the redox system Ce(IV)–isopropyl alcohol. Kinetics and molecular weight. *J. Polym. Sci., Part A: Polym. Chem.* **1989,** *27* (11), 3703-3720.
9. Reddy, G. V.; Umamaheswari, M. V.; Vidya, V.; Krishnaswamy, V., Vinyl polymerisation initiated by ceric ion–methyl cellosolve redox system in aqueous nitric acid. *Polym. Int.* **1994,** *34* (3), 279-287.
10. Das, N. K.; Mandal, B. M., Methylene blue as a retarder of free radical polymerisation: 1. Polymerisation of acrylonitrile, methyl methacrylate and styrene. *Polymer* **1982,** *23* (11), 1653-1658.
11. Haines, R. M.; Waters, W. A., Properties and reactions of alkyl free radicals in solution. Part VIII. The reducing action of some water-soluble radicals. *Journal of the Chemical Society (Resumed)* **1955,** (0), 4256-4260.
12. Mock, W. L.; Shih, N. Y., Structure and selectivity in host-guest complexes of cucurbituril. *The Journal of Organic Chemistry* **1986,** *51* (23), 4440-4446.
13. Freitag, M.; Gundlach, L.; Piotrowiak, P.; Galoppini, E., Fluorescence Enhancement of Di-p-tolyl Viologen by Complexation in Cucurbit[7]uril. *Journal of the American Chemical Society* **2012,** *134* (7), 3358-3366.
